# Modification of amyloid-beta peptide aggregation *via* photoactivation of strained Ru(ii) polypyridyl complexes†

**DOI:** 10.1039/d1sc00004g

**Published:** 2021-04-24

**Authors:** Janaina C. Bataglioli, Luiza M. F. Gomes, Camille Maunoir, Jason R. Smith, Houston D. Cole, Julia McCain, Tariq Sainuddin, Colin G. Cameron, Sherri A. McFarland, Tim Storr

**Affiliations:** Department of Chemistry, Simon Fraser University BC Canada V5A-1S6 tim_storr@sfu.ca; Department of Chemistry and Biochemistry, University of Texas Arlington Texas USA 76019 sherri.mcfarland@uta.edu; Department of Chemistry, Acadia University Wolfville Nova Scotia Canada B4P 2R6

## Abstract

Alzheimer's disease (AD) is a chronic neurodegenerative disorder characterized by progressive and irreversible damage to the brain. One of the hallmarks of the disease is the presence of both soluble and insoluble aggregates of the amyloid beta (Aβ) peptide in the brain, and these aggregates are considered central to disease progression. Thus, the development of small molecules capable of modulating Aβ peptide aggregation may provide critical insight into the pathophysiology of AD. In this work we investigate how photoactivation of three distorted Ru(ii) polypyridyl complexes (**Ru1–3**) alters the aggregation profile of the Aβ peptide. Photoactivation of **Ru1–3** results in the loss of a 6,6′-dimethyl-2,2′-bipyridyl (6,6′-dmb) ligand, affording *cis*-exchangeable coordination sites for binding to the Aβ peptide. Both **Ru1** and **Ru2** contain an extended planar imidazo[4,5-*f*][1,10]phenanthroline ligand, as compared to a 2,2′-bipyridine ligand for **Ru3**, and we show that the presence of the phenanthroline ligand promotes covalent binding to Aβ peptide His residues, and in addition, leads to a pronounced effect on peptide aggregation immediately after photoactivation. Interestingly, all three complexes resulted in a similar aggregate size distribution at 24 h, forming insoluble amorphous aggregates as compared to significant fibril formation for peptide alone. Photoactivation of **Ru1–3** in the presence of pre-formed Aβ_1–42_ fibrils results in a change to amorphous aggregate morphology, with **Ru1** and **Ru2** forming large amorphous aggregates immediately after activation. Our results show that photoactivation of **Ru1–3** in the presence of either monomeric or fibrillar Aβ_1–42_ results in the formation of large amorphous aggregates as a common endpoint, with Ru complexes incorporating the extended phenanthroline ligand accelerating this process and thereby limiting the formation of oligomeric species in the initial stages of the aggregation process that are reported to show considerable toxicity.

## Abstract 2

Photoactivation of a series of Ru(ii) polypyridyl complexes leads to ligand exchange and modulation of amyloid-beta peptide aggregation of relevance to Alzheimer's disease.

## Graphical abstract

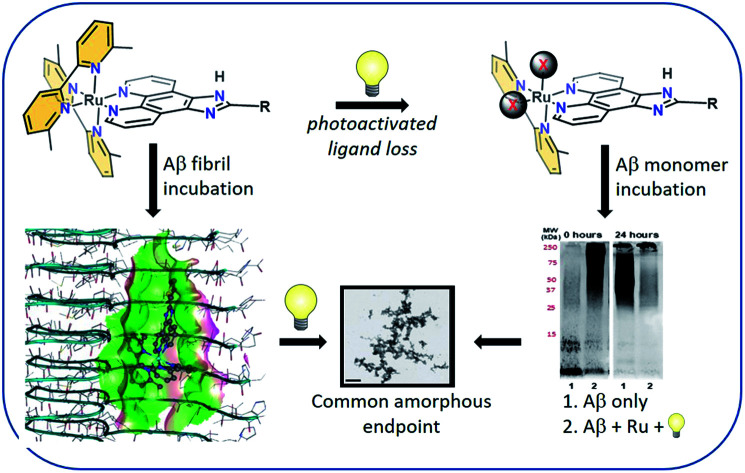

## Introduction

Alzheimer's disease (AD) is the most common form of dementia and is currently the 5^th^ leading cause of death worldwide. An increase in life expectancy is expected to result in a sharp rise in the number of dementia cases in the next 30 years, with approximately 150 million people forecast to be living with dementia by 2050.^1^ The increased incidence of AD, and lack of effective treatment strategies, has stimulated an intense research effort to enhance our understanding of the pathophysiology of this disease and develop new therapeutics. The amyloid hypothesis was first proposed almost 25 years ago, and postulates that the progressive formation of oligomers and aggregates of the Aβ peptide is caused either by increased production or decreased clearance of Aβ, triggering a neurotoxic cascade in the brain.^2–6^ Proteolytic cleavage of the amyloid precursor protein (APP) affords the Aβ peptide in variable lengths (38 to 43 amino acids), with the fragment ending at position 40 (Aβ_1–40_) being the most abundant (∼90%) followed by 42 (Aβ_1–42_, ∼9%).^7^ Truncation at the N-terminus results in Aβ_3(p)-*n*_, Aβ_4-*n*_, and Aβ_11(p)-*n*_ (where p refers to pyroglutamate), which are also components of amyloid plaques.^8–10^ Clinical trials of promising drugs targeting the amyloid pathway have so far failed, either due to off-target effects or a lack of efficacy.^11,12^ There is considerable debate as to when drug treatments should be initiated in AD, and drug trials targeting the amyloid pathway are now focused on healthy people at risk of AD.^13,14^

Oxidative stress is widespread in AD, with early neuronal and pathological changes indicating oxidative damage.^15^ Fenton-type processes involving dysregulated redox-active metal ions (Cu, Fe), and metal-containing Aβ aggregates, are hypothesized to contribute to the production of reactive oxygen species (ROS) and resulting oxidative stress in AD.^16–20^ In addition, metal ion coordination to the Aβ peptide (Fe, Cu, Zn) alters its aggregation pattern, and thus it is hypothesized that metal-ion dysregulation and interaction with Aβ plays a significant role in AD development. A number of approaches for the prevention of metal-ion binding to Aβ have been developed, including the use of metal-binding agents,^21–23^ and metal complexes that target metal-binding residues,^24–27^ thereby modulating peptide aggregation. Due to the multifactorial nature of AD, it is likely that a multifunctional drug development strategy will be needed to effectively treat this disease. Metal complexes capable of modifying the Aβ peptide aggregation process, while also restricting adventitious metal ion binding to Aβ,^28,29^ limiting oxidative stress,^30,31^ inhibiting acetylcholine esterase (AChE) activity,^32^ and initiating peptide cleavage have received considerable attention.^33,34^ Work with Pt complexes has shown that in addition to covalent binding (most likely to Aβ peptide His residues), the incorporation of planar aromatic ligands can enhance non-covalent π–π interactions of metal complexes with the Aβ peptide, providing an additional mechanism to increase targeting.^35–38^ Commonly employed ligands to enhance π–π interactions include 2,2′-bipyridine (bpy), 1,10-phenanthroline (phen), and cyclometalating ligands. Examples include a number of cyclometallated Rh and Ir complexes that have been reported to limit aggregation, and exhibit enhanced emissive properties when bound to the Aβ peptide.^39,40^ Recently, Lim *et al.* reported a series of cyclometallated Ir complexes that promote the photo-induced oxidation of Aβ in the presence of O_2_.^41,42^

Ru(ii) polypyridyl complexes have found widespread application due to their interesting electrochemical, photophysical, and biological properties.^43,44^ In biology, these complexes have shown utility in DNA intercalation^45^ and protein binding.^46^ Ru(ii) polypyridyl complexes can be photoactivated leading to the generation of ROS such as singlet oxygen (^1^O_2_) or ligand dissociation to afford a metal complex capable of binding to biological targets,^47–50^ or the release of biologically active ligands from the metal complex.^51–55^ A number of Ru(ii) polypyridyl complexes have been reported to interact with the Aβ peptide *via* non-covalent π–π interactions. In the case of [Ru(bpy)_3_]^2+^, photoactivation in the presence of the Aβ peptide leads to amino acid oxidation and destabilization of peptide secondary structure *via* generation of ^1^O_2_.^56^ In elegant work, Martí and co-workers have shown that Ru(ii) polypyridyl complexes with a specific extended planar aromatic ligand can also be used as sensitive fluorescent probes for amyloid fibrils^57^ and oligomers.^58,59^ Other Ru(ii) polypyridyl complexes were shown to limit Aβ aggregation, inhibit AChE activity and protect against ROS.^31,32^

**Fig. 1:**
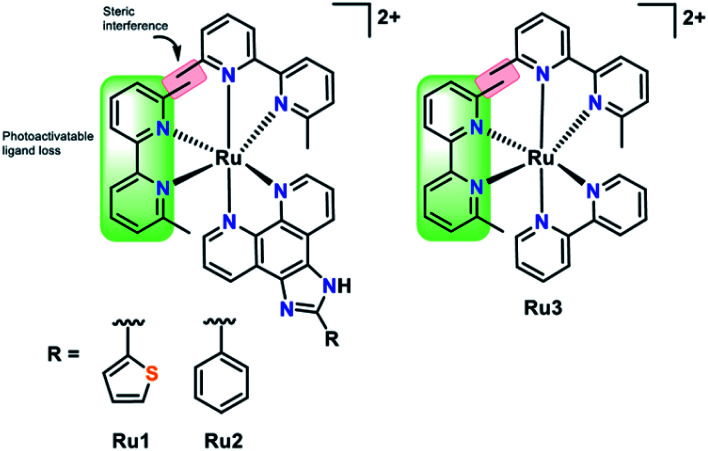
Photoactivatable Ru complexes (**Ru1–3**) used in this work. Visible light photoactivation leads to 6,6′-dimethyl–2,2′-dipyridyl (6,6′-dmb) ligand loss to unmask *cis*-exchangeable coordination sites for Aβ peptide binding.

In this work we have investigated the interaction of a series of strained photoactivatable Ru(ii) polypyridyl complexes with the Aβ peptide (Fig. 1). Photoactivation by visible light leads to loss of a 6,6′-dimethyl-2,2′-dipyridyl (6,6′-dmb) ligand, unmasking *cis*-exchangeable coordination sites capable of binding to biomolecules such as the Aβ peptide. We find that photoactivation is critical for modulating Aβ peptide aggregation, and in addition, the extended planar imidazo[4,5-*f*][1,10]phenanthroline ligand in **Ru1** and **Ru2** enhances Aβ peptide targeting in comparison to the bpy analogue **Ru3**.

## Results and discussion

### Stability and photoactivation

**Scheme 1:**
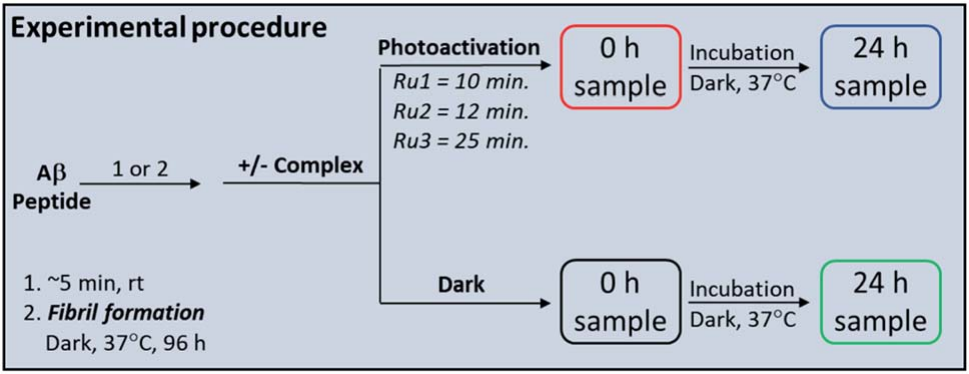
Sample preparation protocol for the Aβ peptide interaction experiments with **Ru1–3**.

Complexes **Ru1** and **Ru2** have been previously reported to dissociate one 6,6′-dimethyl-2,2′-dipyridyl (6,6′-dmb) ligand upon photoactivation,^60,61^ while the photochemical ligand dissociation process for **Ru3** has not yet been reported. In addition, **Ru1** and **Ru2** present limited cytotoxicity in the dark and thus limited potential for off-target toxicity (EC_50_ = 37 μM in HL 60 cell line for **Ru1** and >100 μM in SKMEL 28 cell line for **Ru2**),^60,61^ and these two complexes incorporate a planar aromatic [1,10]phenanthroline ligand, analogues of which have shown high affinity for amyloid aggregates.^57–59,62^ Thus we hypothesized that **Ru1** and **Ru2** would be good candidates for photoactivated binding to the Aβ peptide, while **Ru3** would offer a suitable comparison that does not incorporate the extended [1,10]phenanthroline ligand. The sample preparation protocol for the **Ru1–3** interaction experiments with the Aβ peptide is shown in Scheme 1.

Under our conditions, **Ru1** and **Ru2** were stable in buffer solution over 24 h in the dark, however, **Ru3** exhibited *ca.* 6% loss of a 6,6′-dmb ligand in the absence of light (Fig. S1 and S2†). As expected, **Ru1–3** undergo photochemical ligand dissociation upon exposure to visible light (SOLLA 30W LED, 5.7 mW cm^−2^),^60,61^ with UV-vis measurements indicating reaction completion at 10 min, 12 min, and 25 min, respectively after initial light exposure (Fig. S3†). Although crystal structures and ^3^MC energies are unavailable at this time, it could be the case that the larger π-expansive ligands in **Ru1** and **Ru2** lead to larger distortions in the coordination geometries, which in turn suppresses rechelation and accelerates dissociation times in comparison to **Ru3**. Photoactivation produces very little singlet oxygen (^1^O_2_ yield 0.03 for **Ru1**,^60^ 0.01 for **Ru2**,^61^ 0.01 for **Ru3** ^63^) in comparison to [Ru(bpy)_3_]^2+^ (^1^O_2_ yield 0.56).^64^ The photodissociation was shown to be selective for dissociation of the 6,6′-dmb ligand for **Ru1–3** as demonstrated by ^1^H NMR and ESI-MS (Fig. S4 and S5†). The MS spectra show peaks consistent with different ligands (H_2_O, DMSO, Cl^−^) occupying the coordination sites made available due to 6,6′-dmb photoejection. Interestingly, we observe a decrease in Ru(ii) complex signals in the ^1^H NMR upon photoactivation, and the presence of a precipitate. These results are consistent with the relatively high concentration used in the NMR experiment (200 μM), the number of different complexes formed, and the formation of complexes with limited solubility (Fig. S6†).^65–67^ At lower concentrations (50–60 μM) no precipitate was observed *vide infra*, even after 24 h of incubation, however, at these lower concentrations the ^1^H NMR signals were not discernible. The photoactivation results show the availability of exchangeable coordination sites on **Ru1–3** upon release of the 6,6′-dmb ligand for interaction with the Aβ peptide.

### Binding of **Ru1–3** to the Aβ peptide

**Fig. 2:**
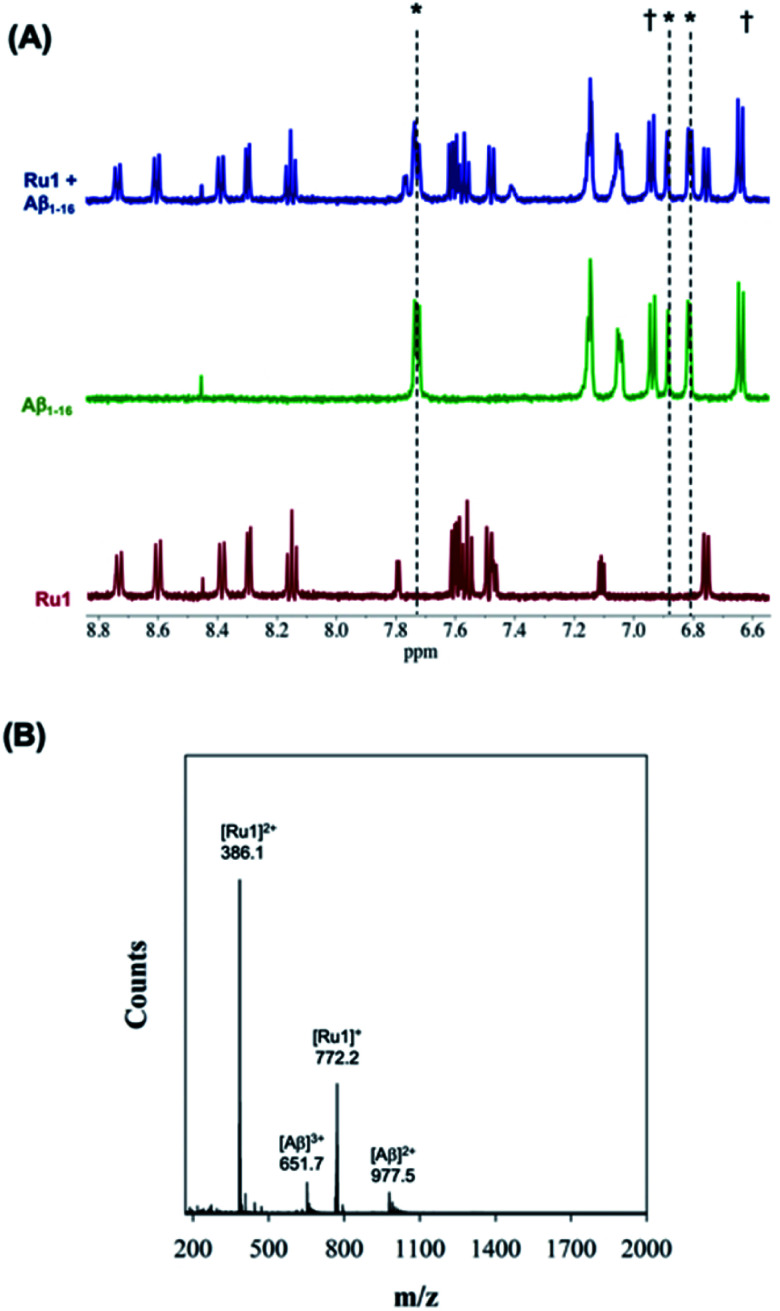
(A) ^1^H NMR spectra of Aβ_1–16_ (200 μM) in the presence of 1.0 eq. unactivated **Ru1** showing no changes of peptide residue signals after 24 h of incubation. Samples were prepared in PBS buffer (0.01 M, pH 7.4) at 37 °C. * His,^6^ His^13^ and His.^14^ † Tyr.^10^ (B) ESI-MS of unactivated **Ru1** + Aβ_1–16_ showing no evidence of adduct formation. Samples were prepared in NH_4_CO_3_ buffer (20 mM, pH 9.0).

We first evaluated the interaction of unactivated **Ru1–3** with the Aβ peptide by ^1^H NMR and ESI-MS. For these initial studies we chose to use the hydrophilic Aβ_1–16_ peptide which includes most of the amino acids associated with metal ion binding (*i.e.* His^6/13/14^), and in addition, has a low propensity to aggregate. Incubation of one eq. of **Ru1–3** with Aβ_1–16_ in the dark over 24 h resulted in no changes in NMR features suggesting no significant interaction in solution (Fig. 2 and S7–S10†).

In addition, the ESI-MS data for unactivated **Ru1–2** in the presence of Aβ_1–16_ show peaks for the intact complexes and the Aβ_1–16_ peptide, and no evidence of adduct formation or a ternary complex under the experimental conditions (Fig. 2 and S9†). Interestingly, while the ^1^H NMR of unactivated **Ru3** with Aβ_1–16_ did not exhibit any changes to peptide residue signals over 24 h (Fig. S8†), the ESI-MS spectrum indicated species consistent with loss of the 6,6′-dmb ligand and adduct formation ([**Ru3**–Aβ_1–16_]^2+^; *m*/*z* = 1197.7) (Fig. S10†). This result is in agreement with the lower stability of unactivated **Ru3** in solution and *ca.* 6% ligand loss measured by ^1^H NMR (Fig. S1†).

**Fig. 3:**
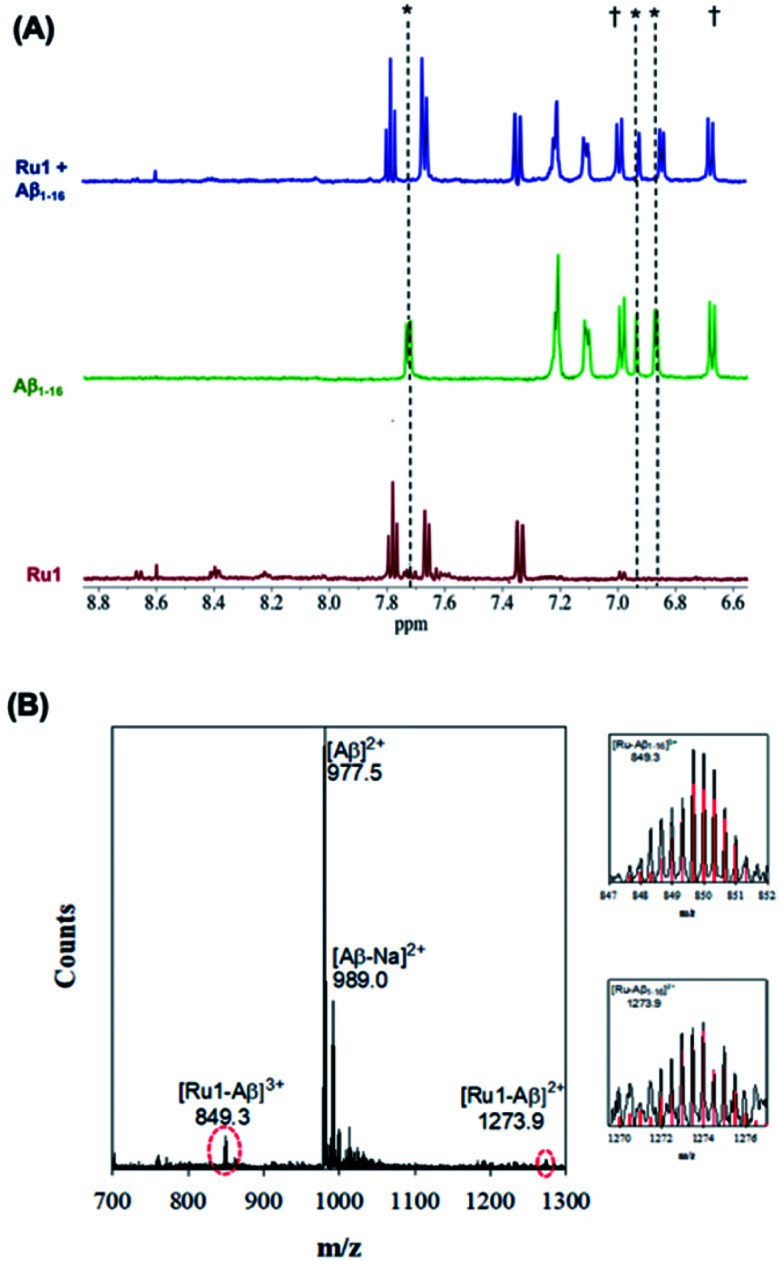
(A) ^1^H NMR spectra of photoactivated **Ru1**–Aβ_1–16_ (200 μM) showing His shifts immediately after photoactivation (10 min.). Samples were prepared in PBS buffer (0.01 M, pH 7.4) at 37 °C. * His,^6^ His^13^ and His.^14^ † Tyr.^10^ (B) ESI-MS of photoactivated **Ru1** + Aβ_1–16_ showing evidence of adduct formation. Zoomed region shows the isotopic pattern of the detected adduct, and in red the theoretical isotopic pattern for the corresponding adduct. Samples were prepared in NH_4_CO_3_ buffer (20 mM, pH 9.0) and data was collected after 10 min of activation.

Upon addition of one eq. of **Ru1** to Aβ_1–16_ and photoactivation for 10 min (SOLLA 30W LED, 5.7 mW cm^−2^) we observed the presence of free 6,6′-dmb in the ^1^H NMR, a shift in select peptide residues, and the loss of signals associated with the Ru(ii) complex (Fig. 3). We attribute the loss of Ru(ii) signals to the presence of multiple species bound to the peptide upon photoactivation and precipitation of the photodissociated complex at the 200 μM concentration. Interestingly, while the majority of the peptide residues do not shift upon photoactivation, the His resonance at 7.78 ppm shifts upfield to 7.71 ppm, overlapping with a free 6,6′-dmb signal at 7.71 ppm with a concomitant increase in integration value (Fig. 3). The data is consistent with binding of an Aβ His residue to photoactivated **Ru1**, and we hypothesize that there is likely no preference for any of the available His residues (His,^6^ His,^13^ His^14^).

Exposure of Aβ_1–16_ alone to the photolysis conditions (SOLLA 30W LED, 5.7 mW cm^−2^) did not shift any of the ^1^H NMR signals in comparison to Aβ_1–16_ in the absence of photolysis. An upfield shift of the His residue at 7.78 ppm is also observed for **Ru2** upon photoactivation in the presence of Aβ_1–16_ (Fig. S7†), and our results are consistent with metal complex – Aβ binding reported for Ru(iii) complexes,^68^ and for Pt(ii) complexes reported by Guo *et al.*^69^ and Hureau *et al.*,^29^ indicating that the His residues are involved in the interaction of **Ru1–2** with Aβ.

While photoactivated **Ru3**–Aβ_1–16_ samples exhibit free 6,6′-dmb ligand, no shifts of any peptide residues were observed after 24 h of incubation, even in the presence of 2 eq. of **Ru3** (Fig. S8†). These results suggest that while Aβ His binding occurs for **Ru1–2**, **Ru3** does not interact with the peptide in the same manner.

The interaction of photoactivated **Ru1–3** with Aβ_1–16_ was further investigated *via* ESI-MS. In contrast to the MS spectrum of unactivated **Ru1**–Aβ_1–16_ (Fig. 2), photoactivated **Ru1**–Aβ_1–16_ indicates the formation of adducts [**Ru1**–Aβ_1–16_]^3+^ (*m*/*z* = 849.3) and [**Ru1**–Aβ_1–16_]^2+^ (*m*/*z* = 1273.9) (Fig. 3). The isotopic distribution confirms the presence of Ru in the adduct peaks, and the masses of the adducts are consistent with loss of the 6,6′-dmb ligand and coordination to the peptide. The ESI-MS data for **Ru2** is similar to that for **Ru1**, indicating adduct formation upon photoactivation of the complex in the presence of the Aβ peptide (Fig. S8†). As detailed above, ESI-MS of unactivated **Ru3** in the presence of Aβ_1–16_ indicates adduct formation, and upon photoactivation a number of adduct peaks are present including ([**Ru3**–Aβ_1–16_]^3+^; *m*/*z* = 798.5), ([**Ru3**–Cl–Aβ_1–16_]^3+^; *m*/*z* = 812.4), and ([**Ru3**–Aβ_1–16_]^2+^; *m*/*z* = 1197.5) (Fig. S10†). The latter peak is also observed in the unactivated ESI-MS spectrum. We also investigated the binding of unactivated and activated **Ru1–3** with the longer length Aβ_1–40_ peptide, and the results are similar to that described for Aβ_1–16_ peptide, showing that adduct formation only occurs for the photoactivated complexes (Fig. S11†). Previous work by Park *et al.* using [Ru(bpy)_3_]^2+^ (ref. 56) and Lim *et al.* using cyclometallated Ir complexes^41,42^ show significant Aβ peptide oxidation upon photoactivation, however, we do not observe evidence of peptide oxidation in the photoactivation experiments by ESI-MS, in agreement with the low ^1^O_2_ quantum yields for **Ru1–3**. Unfortunately, our MS/MS fragmentation experiments were not successful in indicating the residue(s) responsible for peptide binding due to low signal to noise.

It is interesting to note that while **Ru3** exhibits adduct formation in the ESI-MS spectrum, no significant His residue shifts (or any other peptide residue) were observed in the ^1^H NMR experiment. We speculate that while a significant amount of adduct forms in the photoactivation experiments for **Ru1** and **Ru2**, comparatively less adduct forms for **Ru3**. We suggest a potential pre-organizing effect of the extended planar aromatic ligands for **Ru1** and **Ru2**, which facilitates covalent binding upon photoactivation. The enhanced interaction of Aβ peptide aggregates with Ru(ii) polypyridyl complexes incorporating extended planar aromatic ligands has been reported,^57–59,62^ and in addition, planar aromatic ligands enhance covalent adduct formation for Pt(ii) complexes with both the Aβ peptide^57–59,62^ and DNA.^70^

### Influence of **Ru1–3** on Aβ peptide aggregation

**Fig. 4:**
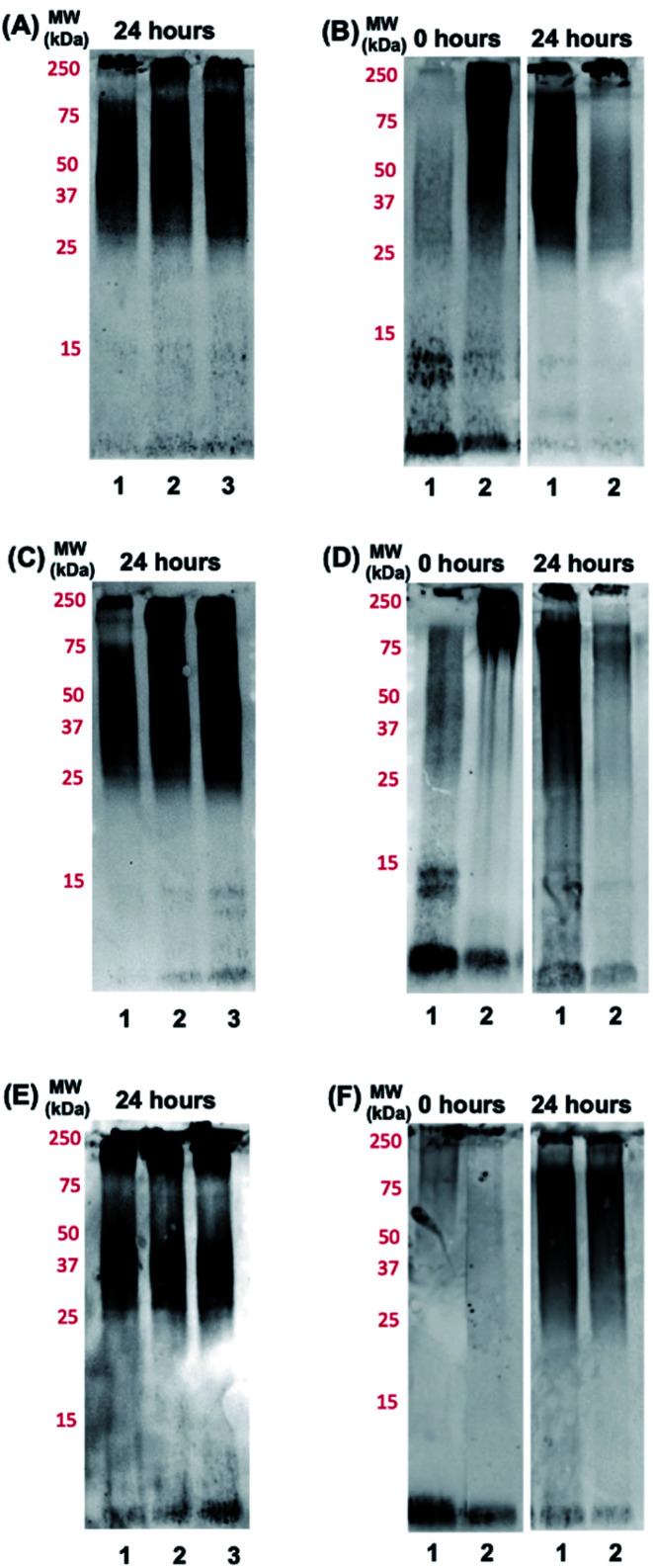
Gel electrophoresis/western blot of Aβ_1–42_ (25 μM) and different concentrations of unactivated **Ru1** (A), **Ru2** (C), and **Ru3** (E) in PBS buffer (0.01 M, pH 7.4) after 24 h of incubation at 37 °C. Lane 1: Aβ_1–42_; lane 2: Aβ_1–42_ + 1.0 eq. Ru(ii) complex; lane 3: Aβ_1–42_ + 2.0 eq. Ru(ii) complex. Influence of photoactivated **Ru1** (B), **Ru2** (D) and **Ru3** (F) on the aggregation profile of Aβ_1–42_. Gel electrophoresis/western blot of 25 μM Aβ_1–42_ and 1.0 eq. of **Ru1–3** in PBS buffer (0.01 M, pH 7.4) at incubation time points 0 h and 24 h, with agitation at 37 °C, using anti-Aβ antibody 6E10. Lane 1: Aβ_1–42_; lane 2: Aβ_1–42_ + 1.0 eq. Ru(ii) complex.

Based on the promising binding data for photoactivated **Ru1–2**, and to a lesser extent **Ru3**, we investigated if the aggregation process of the Aβ peptide could be influenced by the complexes. The Aβ_1–42_ peptide was used in these studies due to the higher propensity for aggregation and toxicity.^71–74^ Using gel electrophoresis/western blotting we determined that unactivated **Ru1–3** (1.0 eq. and 2.0 eq.) did not alter the aggregation pattern of the Aβ_1–42_ peptide (25 μM) relative to peptide alone at 24 h (Fig. 4A, C, and E). Even though a number of Ru(ii) polypyridyl complexes have been reported to interact in a non-covalent manner with the Aβ peptide,^57–59,62^ and in some cases alter the aggregation process,^32,56^ unactivated **Ru1–3** did not exhibit a sufficiently strong interaction to alter the peptide aggregation profile based on the gel electrophoresis experiment.

We next investigated the incubation of the Aβ_1–42_ peptide (25 μM) in the presence of photoactivated **Ru1–3** (0.1 to 2.0 eq.) over 24 h. At 1.0 eq. of **Ru1** and **Ru2**, peptide aggregation is significantly affected, resulting in the formation of higher molecular weight (MW) aggregates *versus* peptide alone, while **Ru3** only shows a similar effect at 2.0 eq. (Fig. S12†). Based on these promising initial results we further studied the effect of the photoactivated complexes (1.0 eq.) on peptide aggregation immediately after photoactivation (0 h) and at the 24 h timepoint. At 0 h, Aβ_1–42_ in the absence of activated Ru complex is primarily present in solution in monomeric and dimeric forms (low MW species), with a range of higher MW species predominating at 24 h in agreement with previous reports (Fig. 4B, D, and F).^33,75,76^ The lack of observable dimer species at 0 h for Aβ_1–42_ alone in Fig. 4F in comparison to 4B and 4D is likely due to slightly different mixing/gel loading times. Photoactivation of **Ru1** and **Ru2** induced the formation of high MW aggregates immediately after photoactivation (*t* = 0 h, Fig. 4B and D), however, **Ru3** did not exhibit induction of high MW species on the gel at the initial timepoint (Fig. 4F). The immediate formation of high MW aggregates for photoactivated **Ru1–2**, as opposed to **Ru3**, suggests that the greater degree of covalent binding observed by ^1^H NMR results in increased peptide aggregation. Interestingly, this immediate change from monomer/dimer to high MW aggregates for **Ru1–2** limits the formation of oligomers in the *ca.* 15–30 kDa range, which are reported to exhibit significant toxicity.^77–79^

After 24 h of incubation, photoactivated **Ru1–2** afford only high MW species (MW > 250 kDa) as observed on the gel (Fig. 4B and D). However, the aggregation pattern for **Ru3** at 24 h appears qualitatively similar to peptide alone (Fig. 4F), again showing a significant difference in comparison to the results for **Ru1–2**. To investigate if the integrity of the peptide was compromised by the presence of the photoactivated Ru complexes (*via* oxidation and/or cleavage),^41,42,56^ a dot blot experiment was performed on the bulk sample (Fig. S13†). The peptide is recognized by the 6E10 antibody at the 24 h timepoint in all cases, showing that high MW aggregates formed in the presence of activated **Ru1–2** do not penetrate the gel, and the lack of observable peptide aggregates on the gel is not due to oxidation/cleavage events restricting interaction with the 6E10 antibody. Overall, the gel electrophoresis results indicate that photoactivation, and covalent binding of **Ru1–2** to the Aβ peptide, are necessary to observe substantial changes in the aggregation pattern, thus highlighting the role of the extended planar aromatic ligands of **Ru1–2** in modulating Aβ peptide aggregation.

In order to further investigate the importance of both photoactivation and the extended aromatic ligand we studied the interaction of the Aβ peptide with the previously reported Ru(ii) polypyridyl complex Ru(bpy)_2_CO_3_,^80^ which incorporates a labile κ^2^-carbonato ligand. Facile ligand exchange of the κ^2^-carbonato ligand provides a Ru(ii) complex with two *cis*-exchangeable coordination sites, similarly to **Ru1–3**. This complex has been used previously to label peptides and proteins in the absence of photoactivation.^81–83^ The ^1^H NMR spectrum of Ru(bpy)_2_CO_3_ in the presence of Aβ_1–16_ did not show a shift of His residues (or any other shift, Fig. S14†), however ESI-MS data showed the formation of a peptide adduct indicating that the complex is able to bind to the peptide, similarly to results for photoactivated **Ru3** (Fig. S15†). In addition, the Aβ_1–42_ peptide aggregation pattern in the presence of 0–2 eq. of Ru(bpy)_2_CO_3_ was investigated by gel electrophoresis and was unchanged in comparison to peptide alone at 0 h and 24 h (Fig. S16†). Thus, the binding and aggregation data for Ru(bpy)_2_CO_3_ are similar to that for photoactivated **Ru3**, providing further support for the importance of the extended hydrophobic [1,10]phenanthroline ligand in **Ru1–2** in facilitating peptide binding and modulating the aggregation pathway.

**Fig. 5:**
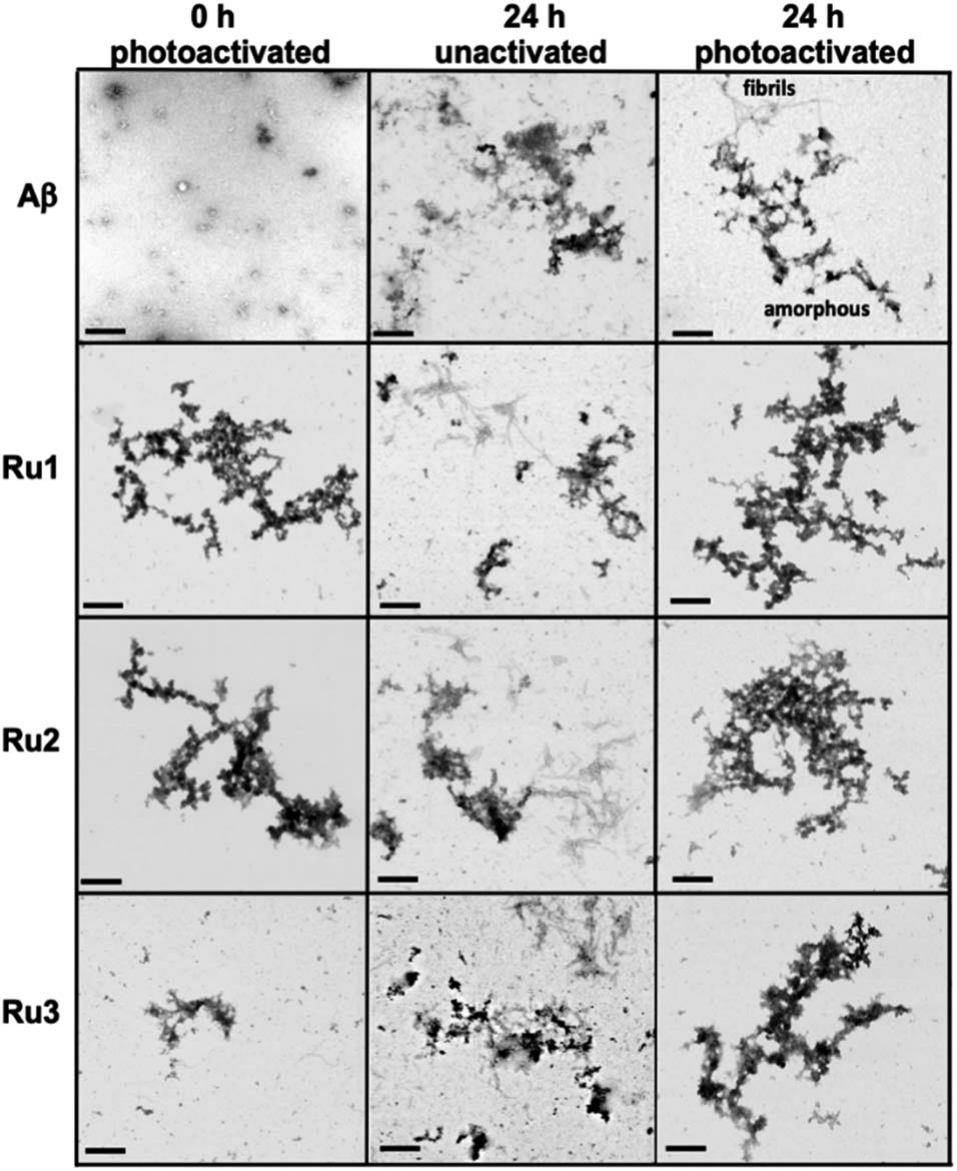
TEM images of the morphology of Aβ_1–42_ aggregates at 0 h (photoactivated samples) and 24 h (for unactivated and photoactivated samples). Conditions: Aβ_1–42_ (25 μM), **Ru1–3** (1.0 eq.) (scale bar 200 nm).

While gel electrophoresis/western blotting revealed the presence of higher MW Aβ_1–42_ species and their size distribution, transmission electron microscopy (TEM) analysis allowed us to characterize larger, Aβ_1–42_ aggregates that are too large to penetrate into the gel matrix. Thus, the combination of these two methods provides a more complete picture of the Aβ_1–42_ aggregation pathway under different conditions.^84,85^ As expected, the TEM images did not show large aggregates for the Aβ_1–42_ sample at 0 h (Fig. 5), however, immediately after light activation, samples containing **Ru1–2** showed large amorphous aggregates, while the sample containing **Ru3** showed the presence of smaller aggregates (Fig. 5). Incubation of Aβ_1–42_ alone for 24 h led to the formation of both large amorphous aggregates and fibrillar species (Fig. 5 and S17†), which agrees with previous reports. Peptide in the presence of unactivated **Ru1–3** showed similar aggregation morphology by TEM as Aβ_1–42_ alone. Incubation of Aβ_1–42_ for 24 h in the presence of photoactivated **Ru1–3** affords similar sized amorphous aggregates (Fig. 5), indicating that the Ru(ii) complexes inhibit fibrillization at 24 h. Our results show that upon photoactivation, **Ru1** and **Ru2** immediately promote changes in peptide aggregation *via* the formation of soluble high MW species and large amorphous aggregates. In contrast, **Ru3** does not promote the formation of large amorphous aggregates immediately, however, similar-sized aggregates are observed by TEM after 24 h (Fig. 5). The TEM images are consistent with the gel electrophoresis results, highlighting that photoactivation is essential for modulation of peptide aggregation.

**Fig. 6:**
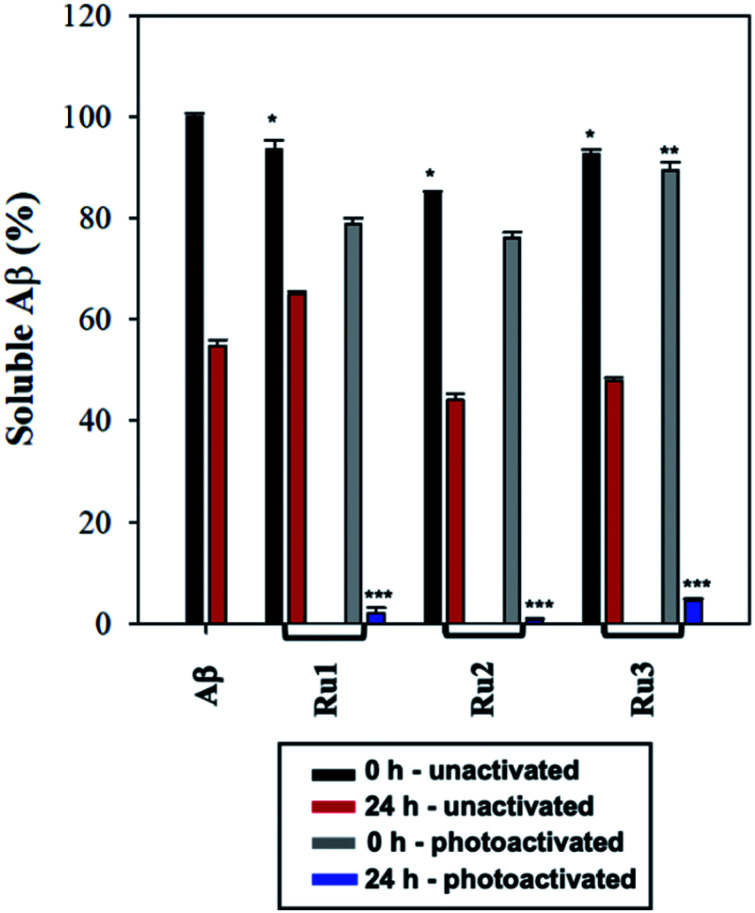
BCA assay of 60 μM Aβ_1–42_ in the presence of 1.0 eq. of **Ru1–3** in PBS buffer (0.01 M, pH 7.4) at 0 h and 24 h with and without photoactivation. Samples were centrifuged at 14 000*g* for 5 min prior to absorbance measurement. Statistically significant difference between: * Aβ_1–42_ 0 h and all three unactivated complexes at 0 h (**Ru1**, *p* = 0.02; **Ru2**, *p* < 0.0001; **Ru3**, *p* = 0.0002); ** photoactivated **Ru1–2** 0 h compared to photoactivated **Ru3** 0 h (**Ru3**, *p* = 0.0003) (no statistical difference between photoactivated **Ru1** and **Ru2** at 0 h); and *** Aβ_1–42_ (24 h) and photoactivated **Ru1–3** (24 h) (*p* < 0.0001). Calculated using 2-way ANOVA.

To further analyze the change in peptide aggregation in the presence of the Ru(ii) complexes, a bicinchoninic (BCA) assay was used to determine the total concentration of Aβ_1–42_ peptide in solution.^86^ Before measurement, the samples were centrifuged to remove insoluble aggregates using an established protocol.^87^ As expected, the results show a *ca.* 50% reduction in soluble peptide after 24 h for peptide alone, which is similar to the change in peptide concentration in the presence of unactivated **Ru1–3** after 24 h (Fig. 6). Immediately after photoactivation, the concentration of soluble peptide is less than peptide alone for samples containing all three complexes, however, **Ru1** and **Ru2** display a slightly larger reduction in Aβ solubility in comparison to **Ru3**, which is consistent with the results obtained from the gel electrophoresis and TEM studies. Strikingly, in the presence of all three activated Ru(ii) complexes, the peptide is almost completely precipitated at 24 h (Fig. 6), which is consistent with the large insoluble amorphous aggregates observed by TEM (Fig. 5), and gel electrophoresis of **Ru1–2** where we observe that the species formed after 24 h incubation does not penetrate the gel (Fig. 4).

Interestingly, the BCA results at 24 h show that photoactivated **Ru3** also significantly decreases peptide solubility, even though the gel electrophoresis experiment still showed soluble high MW species. The morphology of the peptide aggregates formed in the presence of photoactivated **Ru1–3** were similar as indicated by TEM, however we hypothesize that the aggregates formed in the presence of **Ru3** are less stable, and susceptible to partial dissociation in the electrophoresis running buffer (containing 0.1% SDS).

The BCA results show that unactivated **Ru1–3** do not have a large effect on peptide solubility, consistent with the gel electrophoresis and TEM analysis. Immediately after photoactivation, **Ru1–2** lead to a significant change in the aggregation pattern of Aβ_1–42_ as shown by the TEM and gel electrophoresis data. These results highlight the importance of photoactivation for modulation of the peptide aggregation pathway. It is interesting to note that for **Ru3**, upon photoactivation, there is very little change in the aggregation pattern compared to **Ru1–2**, however, at 24 h the change in aggregation for **Ru3** is similar to that for the other two complexes, resulting in large amorphous aggregates and significant precipitation of the peptide from solution.

### Interaction of **Ru1–3** with Aβ peptide fibrils

Based on the ability of the photoactivated Ru(ii) complexes to modulate peptide aggregation in solution we questioned whether the complexes would interact with pre-formed peptide fibrils and if photoactivation would change the morphology/solubility of these ordered insoluble aggregates.

**Fig. 7:**
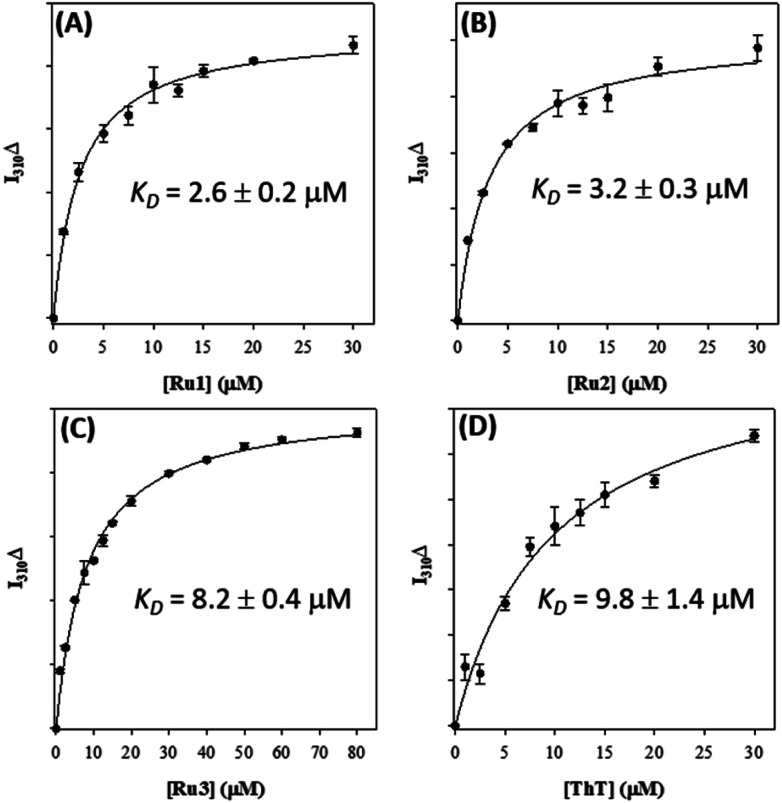
Binding constants of **Ru1** (A), **Ru2** (B), **Ru3** (C), and ThT (D) with pre-formed Aβ_1–42_ fibrils (10 μM in PBS 0.01 M, pH 7.4) measured *via* change in Tyr fluorescence (*λ*_ex_/*λ*_em_ = 275/310 nm).

We first investigated the binding of **Ru1–3** with Aβ_1–42_ fibrils *via* Tyr^10^ fluorescence.^36,88–90^ As expected, **Ru1–3** exhibited negligible photoluminescence in solution (Fig. S18†). To form Aβ_1–42_ fibrils, the monomeric peptide was incubated for 96 h and fibril formation was confirmed by TEM (Fig. S19†). Binding of the Ru complexes was compared to thioflavin T (ThT) as a positive control by employing a single-site binding model (Fig. 7 and S20†).^57,69^ The binding constant of ThT (*K*_d_) under our conditions was determined to be 9.8 ± 1.4 μM, which is in agreement with published values ranging from 5 μM to 11 μM.^91,92^ Using the same protocol, the binding constants for **Ru1** (2.6 ± 0.2 μM), **Ru2** (3.2 ± 0.3 μM), and **Ru3** (8.2 ± 0.4 μM) were obtained for the unactivated complexes (Fig. 7). The values for **Ru1** and **Ru2** compare well to the *K*_d_ of [Ru(bpy)_2_(dppz)]^2+^ (2.1 μM),^57^ while **Ru3** displayed slightly weaker binding to Aβ_1–42_ fibrils. This data shows that the extended planar aromatic ligands for **Ru1** and **Ru2** lead to an enhanced interaction with Aβ_1–42_ fibrils, likely *via* hydrophobic interactions. While the fibril structure is obviously different in comparison to monomeric peptide in solution, the increased potential for hydrophobic interactions between **Ru1–2** and the Aβ_1–42_ peptide may also pre-organize the complexes so that covalent binding occurs more readily to soluble peptide upon photoactivation.

**Fig. 8:**
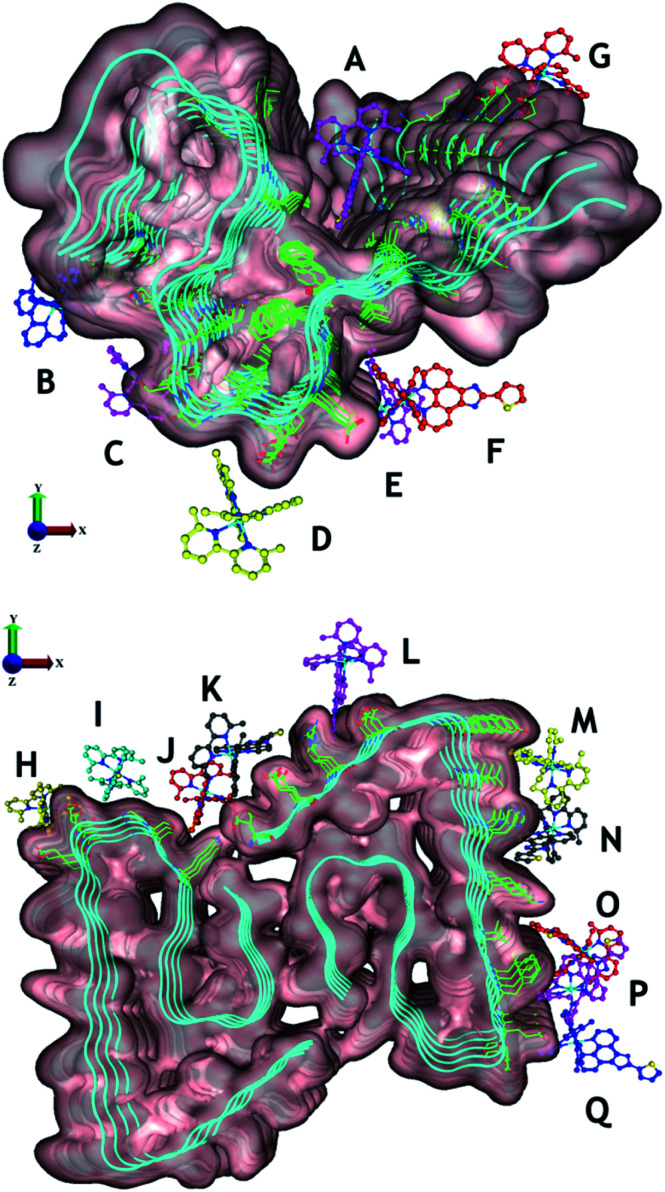
Potential binding sites of unactivated **Ru1–3** to PDB structures 2MXU (top) and 5OQV (bottom). The observed binding sites can generally be characterised as those driven by electrostatic attraction between Ru and polar residues (*e.g.* B [C-terminus], D [Asp^23^], E and F [Glu^22^], G [Glu^11^], H [Glu,^22^ Asp^23^], I [Asp^23^], K [Asp,^1^ Glu^3^], and L [Asp^7^]), and/or driven by phenanthroline–fibril hydrophobic interactions. For clarity, only **Ru1** results are shown with the different complex coloration indicating different binding sites. See Fig. S21–23† for further information on predicted binding interactions for **Ru1–3**. We excluded sites with a ligand interaction (docking score) below 5 kcal mol^−1^.

To gain further insight into the interaction of **Ru1–3** with Aβ_1–42_ fibrils we used molecular docking to visualize potential interactions between **Ru1–3** and Aβ_1–42_ fibrils. We employed protein data bank structures (PDB) 2MXU^93^ and 5OQV^94^ as representative single and double symmetry fibril surfaces, and *via* flexible docking of **Ru1–3** provide further information on the differential interactions of the complexes with Aβ fibrils. The 2MXU structure has a well-defined hydrophobic cleft and 12 β-strand filaments providing sufficient surface area for modeling the interactions with the Ru(ii) complexes, however, the structure does not contain the Aβ_1–10_ region. In contrast, the 5OQV structure includes the complete Aβ_1–42_ peptide, though represents a much shorter length of only 5 strands. Note that as a dimer, binding sites on the other faces were essentially identical and omitted for clarity. In combination, docking with these two structures provides a broad representation of the interaction of **Ru1–3** with Aβ fibrils. Our docking studies show that there is a shallow potential energy surface for fibril binding, with multiple binding sites effectively contributing to the overall binding affinity of **Ru1–3** to the fibrils (Fig. 8 and S21–23†).

Overall, **Ru1–2** are predicted to have a higher binding affinity at a larger number of sites in comparison to **Ru3**, with the relative binding scores detailed in Table S1.† Significant electrostatic interactions between **Ru1–3** and fibril carboxylate residues are predicted (especially at sites B, D, E–I, K, and L), and the more compact **Ru3** complex allows for a closer approach and a more significant interaction at certain sites (*e.g.* sites F, I, O). However, enhanced hydrophobic interactions are predicted for **Ru1–2** containing the extended phenanthroline ligand in comparison to **Ru3**, and these interactions occur at the majority of binding sites on the 2MXU and 5OQV fibril surfaces (Table S1,†Fig. 8 and S23†). The docking results provide further insight into the role of the extended hydrophobic ligands of unactivated **Ru1–2** over **Ru3** in the interaction with the peptide, which may facilitate covalent bond formation once the complexes are photoactivated.

**Fig. 9:**
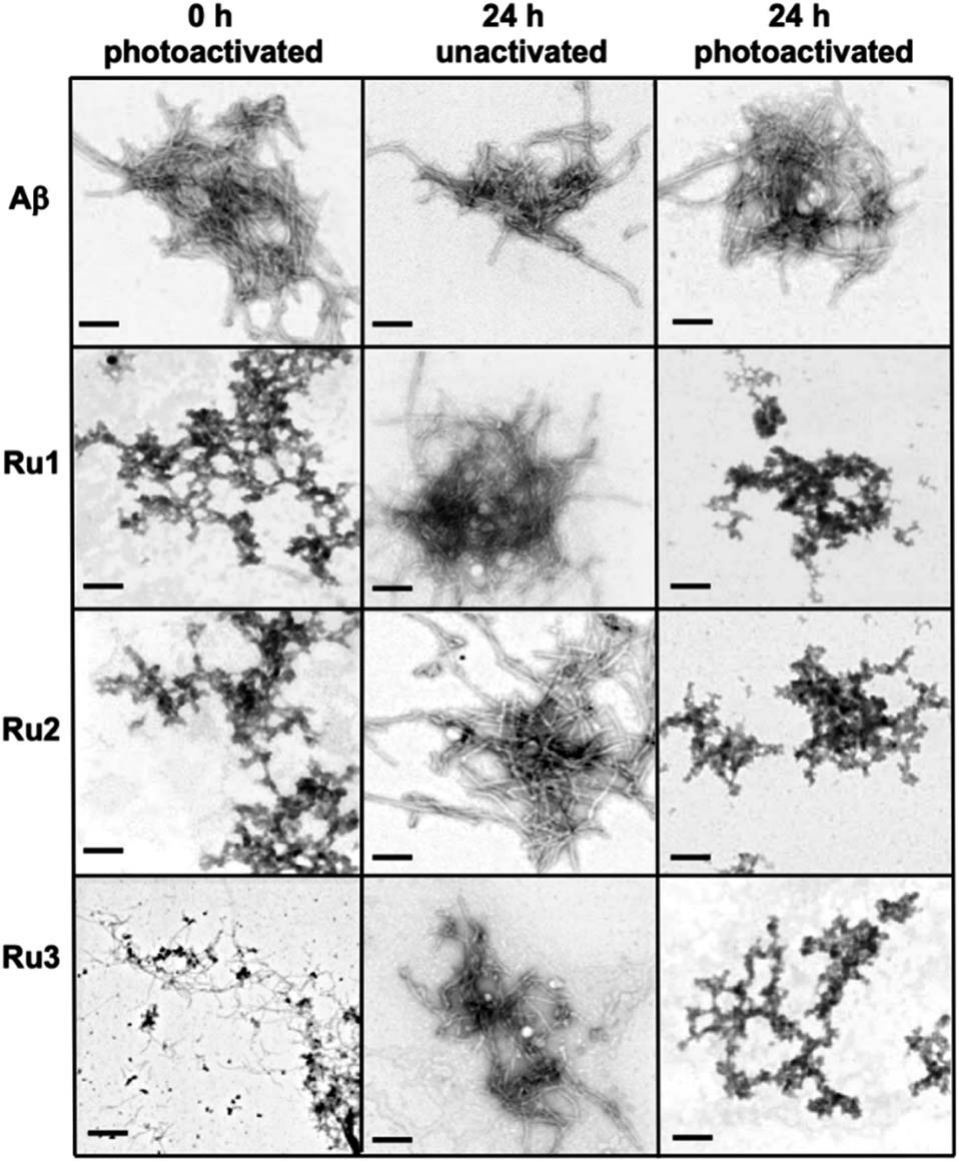
Influence of 1.0 eq. of **Ru1–3** on the morphology of fibrillar Aβ_1–42_ (25 μM) at 0 h (photoactivated samples) and 24 h (for unactivated and photoactivated samples) (scale bar = 200 nm).

In order to investigate if the Ru(ii) complexes could change the morphology of insoluble Aβ_1–42_ fibrils, we incubated the unactivated and photoactivated activated complexes with pre-formed fibrils (see Scheme 1) and monitored for a change in morphology *via* TEM. Our binding studies show that the intact **Ru1–2** complexes have a higher affinity for Aβ_1–42_ fibrils in comparison to **Ru3** (*vide supra*), and thus we hypothesized that photoactivation of the **Ru1–2** complexes (and possibly **Ru3**) may lead to alteration of aggregate morphology. As expected, incubation of Aβ_1–42_ for 96 h exclusively produced mature fibrillar structures (Fig. 9 and S19†). Remarkably, we observed that after addition of the Ru(ii) complexes and photoactivation, an immediate aggregate morphology change from fibrillar to amorphous is observed for **Ru1** and **Ru2**, yet a more gradual change is observed for **Ru3** (Fig. 9). No further changes were observed over an additional 24 h incubation for **Ru1–2**, while for **Ru3** the mixture of amorphous and fibrillar aggregates observed by TEM immediately after photoactivation changes to amorphous at 24 h (Fig. 9 and S24†). Photoactivation is necessary for morphology changes, as there was no difference between pre-incubated Aβ_1–42_ alone and Aβ_1–42_ incubated for additional 24 h in the presence of unactivated **Ru1–3** (Fig. 9). Even though the intact complexes display a high affinity for Aβ_1–42_ fibrils, especially for **Ru1–2** (Fig. 7), the non-covalent interaction does not in itself lead to a change in aggregate morphology.

Upon photoactivation, immediate changes to the peptide aggregate morphology are observed, with **Ru1–2** exhibiting the most significant change in comparison to **Ru3**, in line with the measured binding affinities. However, all three photoactivated complexes exclusively afford amorphous aggregates at the 24 h timepoint.

## Summary

This study demonstrates the ability of photoactivated **Ru1–3** to target and modulate the aggregation pathway of the Aβ peptide. ^1^H NMR showed release of the 6,6′-dmb ligand and His residue shifts for **Ru1** and **Ru2**, indicating that these residues are involved in the binding process. ESI-MS confirmed the release of the 6,6′-dmb ligand upon photoactivation, and also showed the presence of complex–peptide adducts for **Ru1–3**. **Ru1–2**, and to a lesser extent **Ru3**, significantly alter the Aβ_1–42_ aggregation process, with **Ru1–2** promoting the formation of soluble high MW weight aggregates immediately after photoactivation. TEM analysis also shows the formation of large amorphous aggregates for **Ru1–2**, while the aggregates observed for **Ru3** are considerably smaller. This immediate change for **Ru1–2** upon photoactivation limits the formation of low MW peptide oligomers, suggesting that these complexes could bypass the formation of toxic oligomeric species.^77–79^ However, we have not investigated cellular toxicity at this time. After 24 h incubation, photoactivated **Ru1–2** afford very little soluble Aβ_1–42_ as observed in the gel electrophoresis and BCA experiments, and TEM shows formation of large insoluble amorphous aggregates, in comparison to the presence of both fibrils and amorphous aggregates for peptide alone. Photoactivated **Ru3** displays soluble aggregates in the 30–250 kDa size range at 24 h, however, the majority of the peptide has precipitated as indicated by the BCA assay, and large amorphous aggregates are observed by TEM, similarly for **Ru1–2**.

All three Ru(ii) complexes bind to fibrillar Aβ_1–42_, however, **Ru1–2** display a higher affinity and molecular docking studies highlight the importance of the extended hydrophobic ligands in the interaction. Our results indicate that the complexes will be in close proximity to the peptide once the 6,6′-dmb ligand dissociates, likely favoring the formation of a covalent bond between the complexes and peptide. The docking experiments indicate that the extended hydrophobic ligands of **Ru1** and **Ru2** provide for an enhanced interaction with Aβ_1–42_ fibrils, indicating why **Ru3** displays a weaker binding affinity. Extrapolating from the data obtained for **Ru1–3** with fibrillar Aβ_1–42_ and the differences in structures of the complexes, we expect an enhanced interaction of **Ru1–2** with the monomeric peptide in comparison to **Ru3**. Indeed, a number of similar Ru(ii) complexes have been shown to interact with oligomeric species and not just fibrils.^58,59^ TEM images also demonstrated that the complexes are able to modify the morphology of mature fibrils after photoactivation, generating insoluble amorphous aggregates, with **Ru1–2** able to induce this change much more quickly in comparison to **Ru3**.

In this proof-of-principle study we show that photoactivation of **Ru1–3** is critical for modulating the Aβ_1–42_ aggregation process. The formation of amorphous aggregates in the presence of the photoactivated Ru(ii) complexes is a common endpoint, either starting with monomeric peptide or fibrils. While visible light is incompatible with external activation of **Ru1–3** due to limited tissue penetration, the recent development of near-infrared photoactivatable Ru complexes may provide an opportunity for this strategy in AD treatment moving forward.^95–97^ Overall, our results show that the extended hydrophobic ligands present in **Ru1** and **Ru2** enhance the peptide interaction, especially at early time points, facilitating the formation of a covalent adduct between the Ru(ii) complexes and Aβ when samples are photoactivated.

## Author contributions

JCB, SAM, and TimS designed the research project. JCB, LMFG, CM, JRS, HDC, JM, TariqS, and CGC completed experiments. JCB, JRS, SAM, and TimS analyzed data and wrote the manuscript.

## Conflicts of interest

S. A. M. has a potential research conflict of interest due to a financial interest with Theralase Technologies, Inc. and PhotoDynamic, Inc. A management plan has been created to preserve objectivity in research in accordance with UTA policy.

## Supplementary Material

SC-012-D1SC00004G-s001
