# Supplementary material for: Modification of amyloid-beta peptide aggregation via photoactivation of strained Ru(ii) polypyridyl complexes
Source: Chem Sci. 2021 Apr 24;12(21):7510–20. doi: 10.1039/d1sc00004g (PMC8171320; doi:10.1039/d1sc00004g)
Supplement: SC-012-D1SC00004G-s001 [file SC-012-D1SC00004G-s001.pdf]

Electronic Supplementary Material (ESI) for Chemical Science.
This journal is © The Royal Society of Chemistry 2021

SUPPORTING INFORMATION

Modification of Amyloid-beta Peptide Aggregation *via* Photoactivation of Strained Ru(II) Polypyridyl Complexes

Janaina C. Bataglioli,[a] Luiza M. F. Gomes,[a] Camille Maunoir,[a] Jason R. Smith,[a] Houston D. Cole,[b] Julia McCain,[c] Tariq Sainuddin,[c] Colin G. Cameron,[b] Sherri A. McFarland,[b*] and Tim Storr[a*]

[a]Department of Chemistry, Simon Fraser University, BC, Canada, V5A-1S6

[b]Department of Chemistry and Biochemistry, University of Texas, Arlington, Texas, USA, 76019

[c]Department of Chemistry, Acadia University, Wolfville, Nova Scotia, Canada, B4P 2R6

Experimental Information:

All common reagents were purchased from commercial suppliers and used without further purification. All Ru(II) complexes, **Ru1**, **Ru2**, and, **Ru3** were synthesized by the McFarland group.[1, 2, 3] The Aβ_{1-16} peptide was purchased from Genscript (Piscataway, NJ, USA), and Aβ_{1-42} and Aβ_{1-40} from 21st Century Biochemicals (Malborough, MA, USA) and they were all monomerized before use according to a reported procedure.[4, 5] Aβ_{1-16} was dissolved in double distilled water (ddH_2O), while A$\beta_{1-40/1-42}$ was dissolved in DMSO and ddH_2O in a 1:1 mixture, unless stated otherwise. The stock peptide solution concentration was determined by absorbance with the use of a Thermo Nicolet UV nanodrop of Tyr[10] considered as free tyrosine (extinction coefficient of 1410 M^{-1} cm^{-1} at 280 nm).[6] UV-Vis spectra were obtained on a Cary 5000 spectrophotometer. ^{1}H NMR spectra were recorded on a Bruker AV-600 instrument. TEM images were obtained using an OSIRIS FEI scanning TEM (STEM) operating at 200 kV. ESI-MS experiments were performed on an Agilent 6130 mass spectrometer connected to an Agilent 1260 HPLC system. The emission spectra and determination of the binding affinities was performed on

a Fluorolog-3® fluorimeter. Default parameters were used for all computational procedures unless stated otherwise.

1.1.1. Photoejection of 6,6'-dimethyl-2,2 Bipyridine (6,6'-dmb) Ligand

Ru(II) complexes were dissolved in DMSO and added to a phosphate buffered saline solution (PBS, 0.01 M Na_2HPO_4, 0.001 M KH_2PO_4, 0.14 M NaCl, 0.003 M KCl, pH 7.4). Photoejection experiments were carried out *via* UV-Vis using a visible light source with cool white colour (5500 – 6000 K) (SOLLA 30W LED). Data were collected from 200-900 nm, and irradiation intervals were as short as 1 min. at early times and after 15 min., data were collected every 5 min. until 60 min. of experiment. The photoejection time was determined when no further spectral changes were observed. Photoejection kinetics were analyzed by plotting the normalized change in absorption at two wavelengths against irradiation time using a published method.[7, 8, 9, 10] The wavelength selected were those within 50 nm of the longest wavelength isosbestic point and exhibited the greatest change in the course of the experiment.

1.1.2. ^{1}H NMR Binding Assay of $A\beta_{1-16}$ Peptide to Ru(II) Complexes

Deuterated PBS (0.01 M, pH 7.4) buffer was prepared by removal of water by vacuum drying of PBS buffer and dissolving the powder in D_2O. $A\beta_{1-16}$ and Ru(II) stock solutions in DMSO (1 mM) were dissolved in deuterated PBS (0.01 M, pH 7.4) buffer, and the ^{1}H NMR spectra of $A\beta_{1-16}$ alone, Ru(II) complexes (200 μM - kept in the dark or exposed to light for their respective activation time), and $A\beta_{1-16}$ plus Ru(II) complexes (1:1 eq. dark or exposed to light for their respective activation time) were collected after solubilization at 0 h and 24 h.

1.1.3. Mass Spectrometry of Binding of Aβ Peptide to Ru(II) Complexes

Samples were analyzed by direct infusion (1 – 4 μL) of analyte into a mobile phase of 1:1 water:acetonitrile containing 5 mM ammonium acetate (pH unmodified), flowing at 0.3 mL/min and maintained at 30 °C. All components of the mobile phase were MS grade and water was ultra-pure grade from MilliQ A-10 system. Nitrogen drying gas was heated to 250 °C and run at 5 L/min with a nebulizing pressure of 15 psig. Voltages were: capillary 3 kV, fragmentor 175 V, skimmer

30 V, octupole 250 V. Samples were prepared as ~1 mg/mL of total protein (A$\beta_{1\text{-}16/1\text{-}40}$) in ammonium carbonate (0.02 M, pH 9) buffer with 0 or 1 eq. of Ru(II) complexes (dark and activated).

MS/MS studies were undertaken with Synapt G2-Si High Definition hybrid quadrupole (Q)-traveling wave (T-wave) ion mobility (TWIMS)-time-of-flight (TOF) mass spectrometer (Waters Corp., Milford, MA, USA) equipped with an electrospray ionization (ESI) source. ESI conditions were positive ionization mode; voltage 3.0 kV; cone voltage 20 V; source offset 50 V; source temperature 100 °C; desolvation temperature 200°C; cone gas flow 30 L.h-1; desolvation gas flow 600 L.h-1. The sample (150 μM 1:1 A$\beta_{1\text{-}16}$: activated **Ru1-3** initially) was directly infused into the mass spectrometer and the MS/MS experiments were performed using Δm/z 50-1500 and scan rate 0.25 Hz. Data were post-acquisition lock-mass corrected using leucine-enkephalin solution at a concentration of 2.0 μg.mL^{-1}.

1.1.4. Gel Electrophoresis and Western Blotting

Lyophilized A$\beta_{1\text{-}42}$ was dissolved in 1:1 DMSO/ddH_2O to obtain a stock solution with a concentration of approximately 250 μM. The A$\beta_{1\text{-}42}$ stock solution was diluted to 25 μM in PBS (0.01 M, pH 7.4) then incubated at 37 °C with continuous agitation at 200 rpm to form aggregates in the presence of activated and non-activated Ru(II) complexes. For the first set of experiments the peptide was incubated for a total of 24 h, in the presence of different concentrations of Ru(II) complexes (0.10, 0.25, 0.50, 1.0, 1.5, and 2.0 eq.). For the second set of experiments, 1.0 eq. of Ru(II) complexes was also incubated for a total of 24 h, but aliquots were collected at different time points (0 h and 24 h). Electrophoresis separation of peptide aggregates was completed using 8-16% Mini-PROTEAN® TGX Precast Gels from Bio-Rad, at 100 V for 100 min in running buffer (25 mM Tris, 192 mM glycine, 0.1% SDS). The gels were then transferred to a nitrocellulose membrane for 1 h at 100 V at 4 °C, followed by blocking of the membrane in a 3% BSA solution in Tris-buffered saline (TBS) (0.02 M Tris, 0.15 M NaCl, 0.003 M KCl) for 1 h. The membrane was incubated in a solution (1:2000 dilution) with a primary antibody that recognizes Aβ, 6E10, (Biolegends) overnight. After washing 5 x 5 min with TBS, the membrane was incubated in a solution containing the secondary antibody (Horseradish peroxidase, Caymen

Chemicals) for 3 h. A Thermo Scientific SuperSignal® West Pico Chemiluminescent Substrate kit was used to visualize the Aβ species using a Bio-Rad ChemiDoc™ MP imaging system.

1.1.5. Transmission Electron Microscopy (TEM)

TEM grids were prepared from the 1:1 Ru(II)/Aβ samples from the Western blot assay after 0 h and 24 h incubation for the monomeric form of $A\beta_{1-42}$ and 0 h and 24 h incubation at 37 °C after 96 hours of peptide incubation for the fibrillar form of $A\beta_{1-42}$. TEM grids were prepared following previously reported methods.[11, 12] In order to increase hydrophilicity of the Ultrathin Carbon Film 400-mesh grids (Ted Pella), the grids were glow discharged in a vacuum for 10 seconds. Drops of samples (10 μL) were placed onto a sheet of parafilm and the TEM grid was placed on the drop for 5 min. The grid was then placed on top of syringe-filtered 5% uranyl acetate for 1 min. Excess uranyl acetate was removed using a tissue between drops. The grid was allowed to air-dry for at least 15 min. Bright field images were obtained on a FEI Tecnai Osiris STEM at 200 kV.

1.1.6. Binding Constant (K_d)

$A\beta_{1-42}$ film was dissolved in 1:1 DMSO/ddH_2O, and the stock solution was diluted to a final concentration of 10 μM in PBS (0.01 M, pH 7.4) buffered solution and incubated for a period of 96 h at 200 rpm at 37 °C. Separate Aβ solutions were prepared with the Ru(II) complexes (0.10, 0.25, 0.50, 0.75, 1.0, 1.25, 1.5, and 2.0 eq., and up to 8 eq. for **Ru3**) and the fluorescence intensity was measured immediately ($\lambda_{ex}/\lambda_{em}$ = 275/310 nm) to minimize the effects of ligand dissociation / covalent binding. Dissociation constants were determined using a single-site binding model as reported.[13] Analysis of the ThT fluorescence response (Figure S15D) by the same single-site binding model ($\lambda_{ex}/\lambda_{em}$ = 275/480 nm) affords $K_d = 10.6 \pm 1.0$ μM which is in agreement with the same analysis of the Tyrosine response $K_d = 9.8 \pm 1.4$ μM.

1.1.7. BCA Assay

$A\beta_{1-42}$ (60 μM) was incubated in PBS buffer (0.01 M, pH 7.4) with and without the Ru(II) complexes (1 eq.) for a period of 24 h for the BCA assay. The samples were centrifuged at 14,000

g for 5 min., and aliquots were taken at 0 h and kept in the freezer. In a 96 well plate, 20 μL of solution were added in triplicate for each time point, and 200 μL of working reagent from the Thermo Fisher BCA Protein Assay® kit was added to each well. The plate was then incubated for 30 min. at 37 °C, and the concentration of peptide in the supernatant was analyzed by measuring the absorbance at 562 nm.

1.1.8. Docking

All molecular mechanics methods were performed in the Molecular Operating Environment version 2015-19 (MOE, Chemical Computing Group, Montreal, Canada) using the Born solvation model (LJ 12-6, dielectrics 78.6 and 4.0). All DFT calculations were performed in Gaussian 16 (G16RevC.01)[14] using the polarizable continuum model (PCM, water) for solvation. Docking was performed on the whole surface of each PDB structure. Trios of sidechains of the fibril structures form channels perpendicular to the axis of growth, and each of these channels was defined as a binding site for docking. For each ligand, 1, 000 initial binding poses were generated per channel, with grid-based energy minimization (GRIDMIN). The London_dG scoring function was used to select the best 300 poses for energy minimization (RMSG=0.01, with fixed backbone atoms and unrestrained side chains). Results were ranked based on the forcefield interaction energy score. Ligands were not restricted from moving across different channels.

Prior to undertaking docking, our force fields were investigated for their ability to reproduce correct ligand structures. Due to its ubiquity in biological processes, Fe is considerably better parameterized than Ru. We thus looked at both the Fe(II) and Ru(II) compounds, as the properties of the ligand complexes would be largely identical within the limits of molecular mechanics. We compared AMBER-AM1-BCC with MMFF94x in a Born solvation model, using DFT (B3LYP/LANL2DZ in PCM continuum solvent model) as our reference.

The AMBER force field was originally developed to reproduce the thermodynamic properties of proteins, nucleic acids, and some small molecules, and as such is a good representation of the geometry and electrostatics of the protein and especially non-bonded interactions.[15] It has the additional advantage of being compatible with the semi-empirical quantum mechanics method known as AM1-BCC for small molecules, and as such has the capacity to reasonably well describe electrostatics of small molecules.[16] We additionally looked at the

MMFF94x force field. A force field updated from the original MMFF94 and developed to describe drug-like molecules, the specific implementation within MOE has the additional benefit of being able to reasonably guess the properties of small molecules and metals which it wasn't originally parameterized for.[17] Thus, MMFF94x has the advantage of potentially being more accurate in reproducing the geometry and electrostatics of small molecules, but the disadvantage of not being directly developed for describing protein interaction energies. Even so, the use of MMFF94x for docking purposes has previously been described and benchmarked.[18, 19, 20]

Figure S25 demonstrates that MMFF94x performs considerably better at reproducing the DFT structures for both metal centres. We therefore performed our docking using the Ru(II) complexes and the MMFF94x force field.

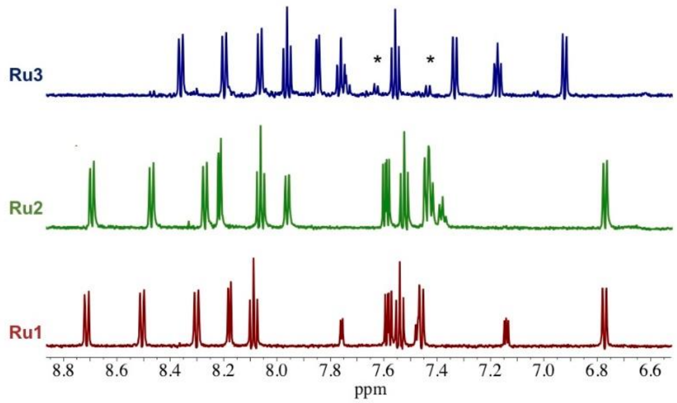

Figure S1. ^{1}H NMR spectra showing the absence of 6,6'-dmb free ligand after 24 h of incubation of unactivated **Ru1** (red) and **Ru2** (green) (200 μM) and an indication of free 6,6'-dmb ligand for unactivated **Ru3** (blue) (200 μM) in phosphate buffered solution (PBS) (0.01 M, pH 7.4). * 6,6'-dmb ligand.

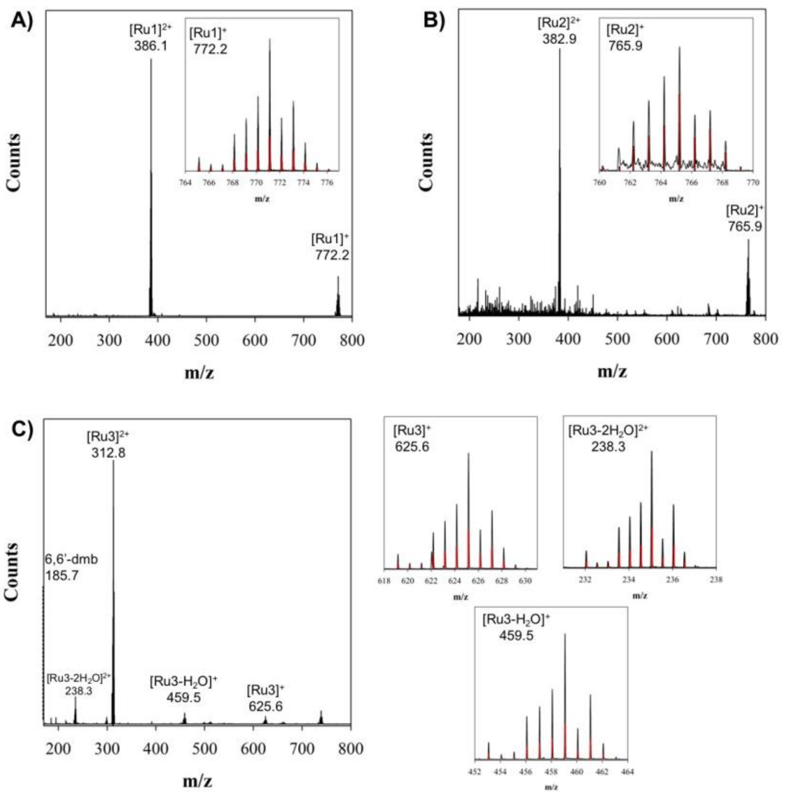

Figure S2. ESI-MS of unactivated **Ru1** (A), **Ru2** (B), and **Ru3** (C) in NH_4CO_3 buffer (20 mM, pH 9.0) showing the stability of **Ru1-2** when kept in the dark and ligand exchange for **Ru3**. Zoomed in regions show the isotope pattern for Ru complexes, red shows the theoretical isotope pattern expected for the complexes.

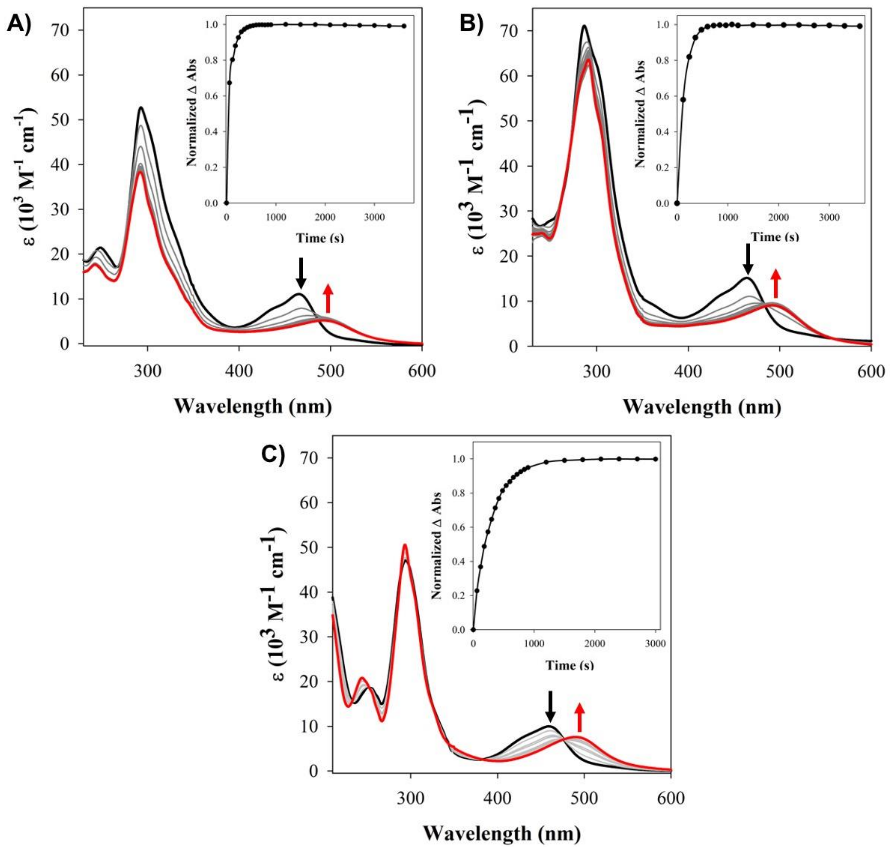

Figure S3. Photochemical ligand dissociation of **Ru1** (A), **Ru2** (B), and **Ru3** (C) (10 μM) in PBS buffer (0.01 M, pH 7.4) monitored by UV-Vis absorption spectroscopy. Data were collected first for unactivated samples (black spectra). Photoactivation was followed at 1 minute intervals up to 15 min., and then every 5 min. (grey spectra) until completion of experiment at 60 min. (red spectra). Insets show the change in absorption at 486 nm (**Ru1**), 483 nm (**Ru2**), and 477 nm (**Ru3**), with complete release of the ligand in 10 min., 12 min., and 25 min. for **Ru1**, **Ru2**, and **Ru3** respectively.

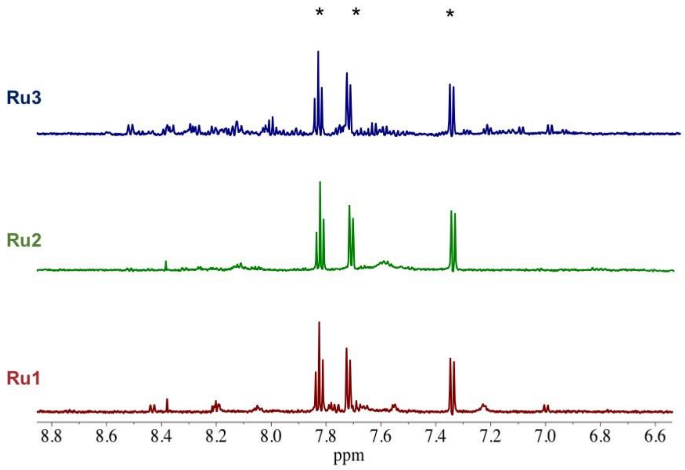

Figure S4. ^{1}H NMR spectra showing the presence of free 6,6'-dmb ligand immediately after activation of **Ru1** (red), **Ru2** (green), and **Ru3** (blue) (200 μM) in PBS buffer (0.01 M, pH 7.4). * 6,6'-dmb ligand.

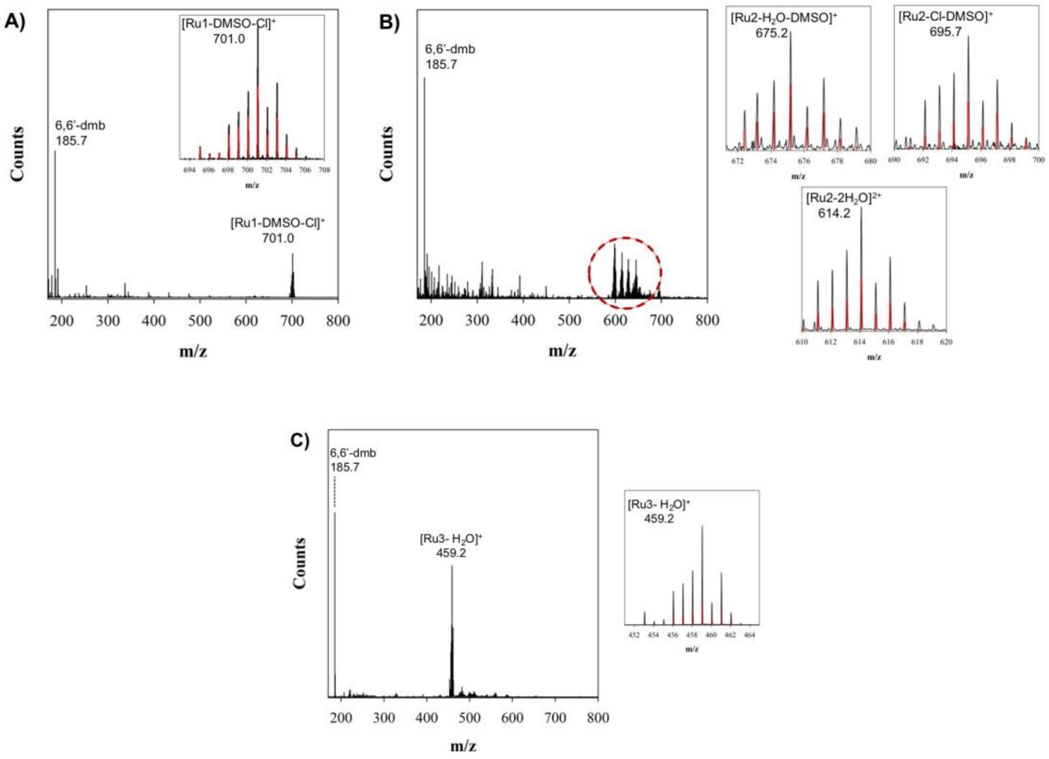

Figure S5. ESI-MS of activated **Ru1** (A), **Ru2** (B), and **Ru3** (C) in NH_4CO_3 buffer (20 mM, pH 9.0) showing the release of the 6,6'-dmb ligand and new ligands occupying the vacant sites of the complexes. Zoomed in regions show the isotope pattern for Ru complexes, red shows the theoretical isotope pattern expected for the complexes.

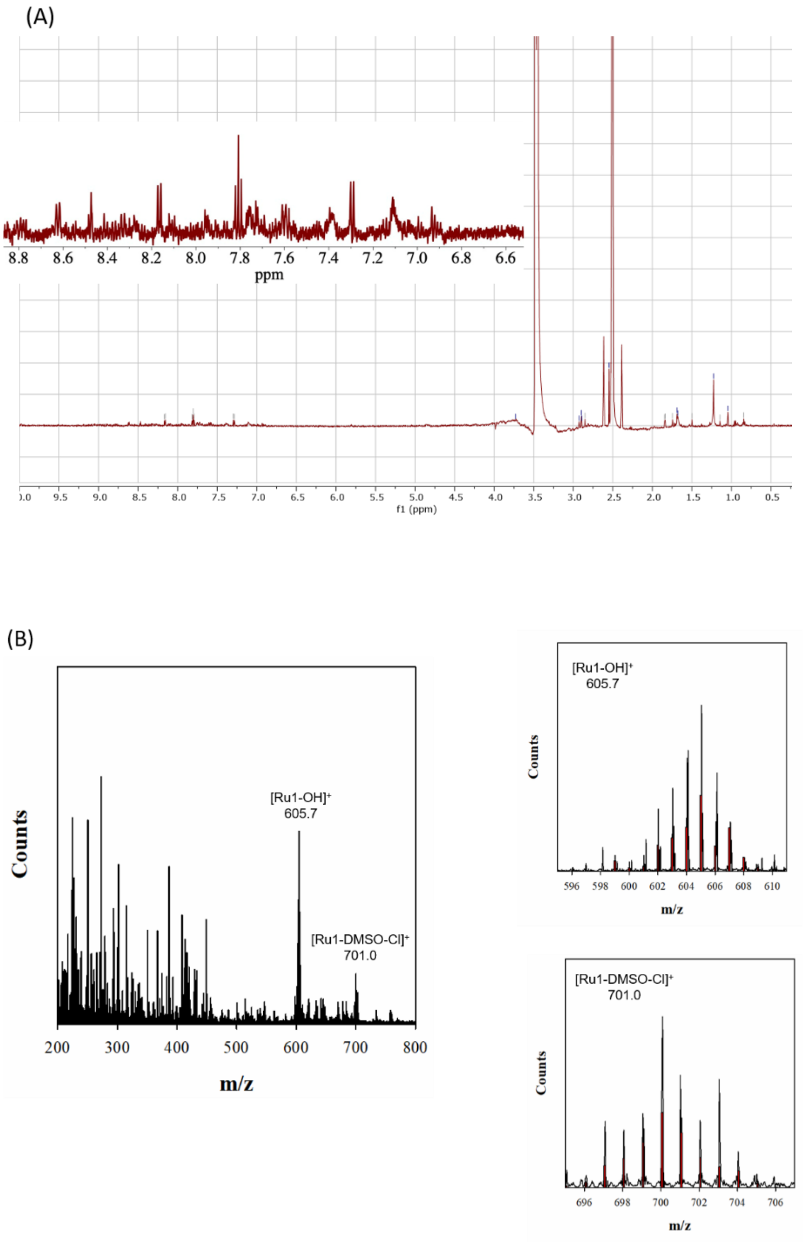

Figure S6. (A) ^{1}H NMR of the red precipitate from photoactivation of **Ru1** in 5% DMSO-d and PBS buffer (0.01 M, pH 7.4). (B) ESI-MS of the red precipitate from photoactivation of **Ru1** showing the release of the 6,6'-dmb ligand and new ligands occupying the vacant sites of the complexes. Zoomed in regions show the isotope pattern for Ru complexes, red shows the theoretical isotope pattern expected for the complexes.

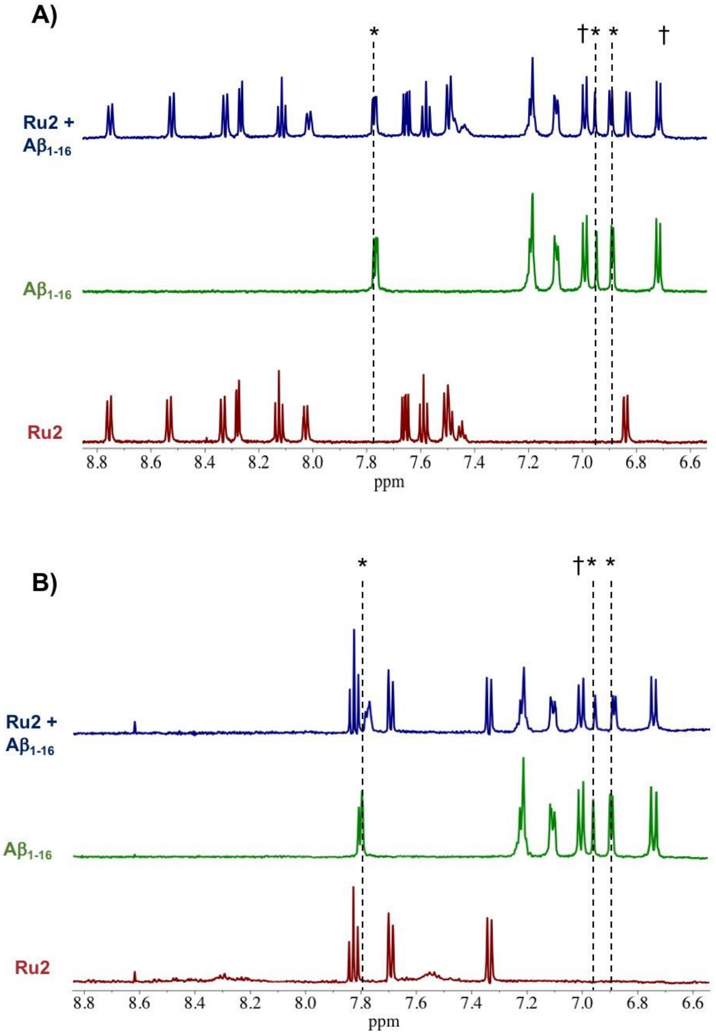

Figure S7. (A) ^{1}H NMR spectra of Aβ_{1-16} (200 μM) in the presence of 1.0 eq. unactivated **Ru2** showing no changes of peptide residues after 24 h of incubation. (B) ^{1}H NMR spectra of photoactivated **Ru2**-Aβ_{1-16} (200 μM) showing His shifts immediately after photoactivation time (12 min.). Samples were prepared in PBS buffer (0.01 M, pH 7.4) at 37 °C. * His^{6}, His^{13} and His^{14}. † Tyr^{10}.

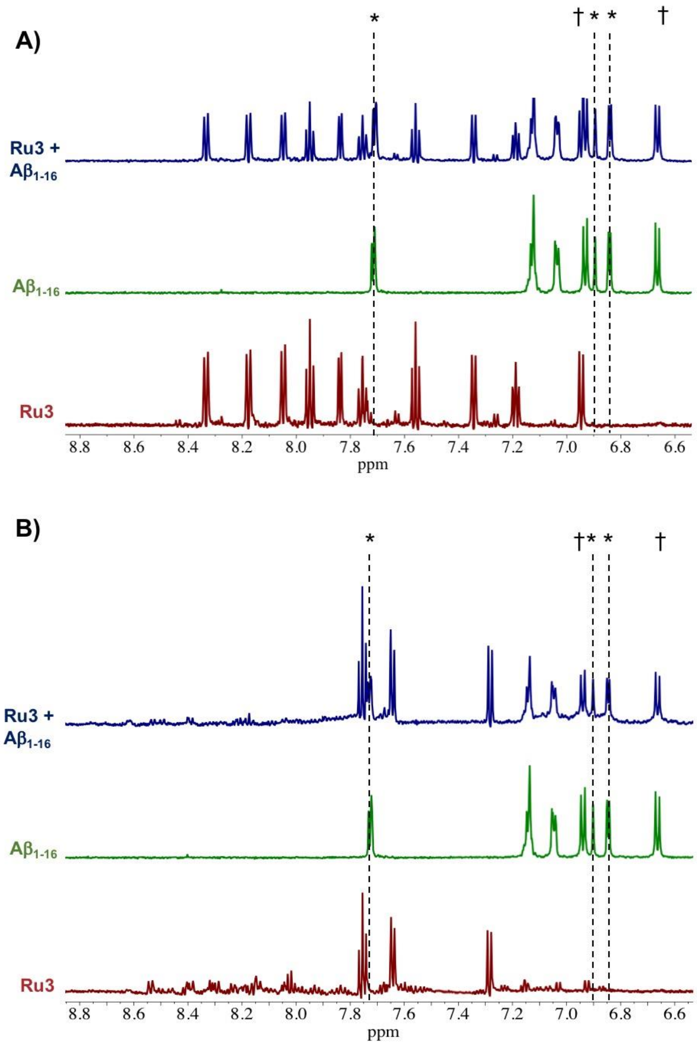

Figure S8. (A) ^{1}H NMR spectra of A$\beta_{1\text{-}16}$ (200 μM) in the presence of 1.0 eq. unactivated **Ru3** showing no changes of peptide residues after 24 h of incubation. (B) ^{1}H NMR spectra of photoactivated **Ru3**-A$\beta_{1\text{-}16}$ (200 μM) showing His shifts immediately after photoactivation time (25 min.). Samples were prepared in PBS buffer (0.01 M, pH 7.4) at 37 °C. * His^6, His^{13} and His^{14}. † Tyr^{10}.

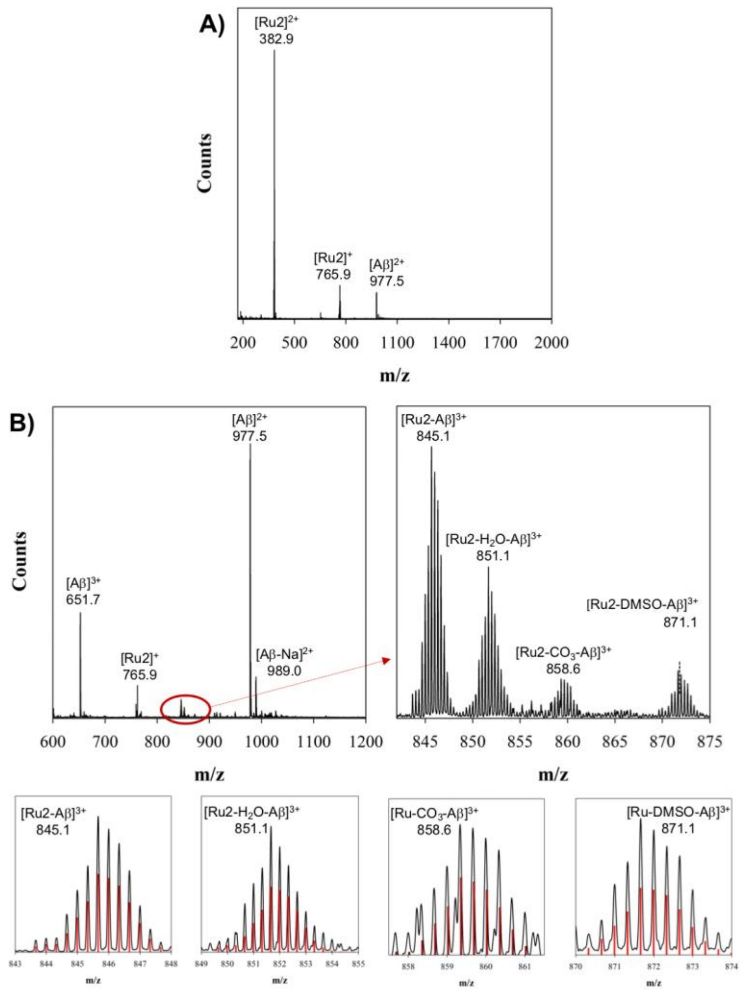

Figure S9. (A) ESI-MS of unactivated **Ru2** + A$\beta_{1\text{-}16}$ showing no evidence of adduct formation. (B) ESI-MS of photoactivated **Ru2** + A$\beta_{1\text{-}16}$ showing evidence of adduct formation. Zoomed regions exhibit the isotopic pattern of the adducts detected, and in red the theoretical isotopic pattern for the corresponding adduct. Samples were prepared in NH_4CO_3 buffer (20 mM, pH 9.0) and data was collected after 12 min. of activation.

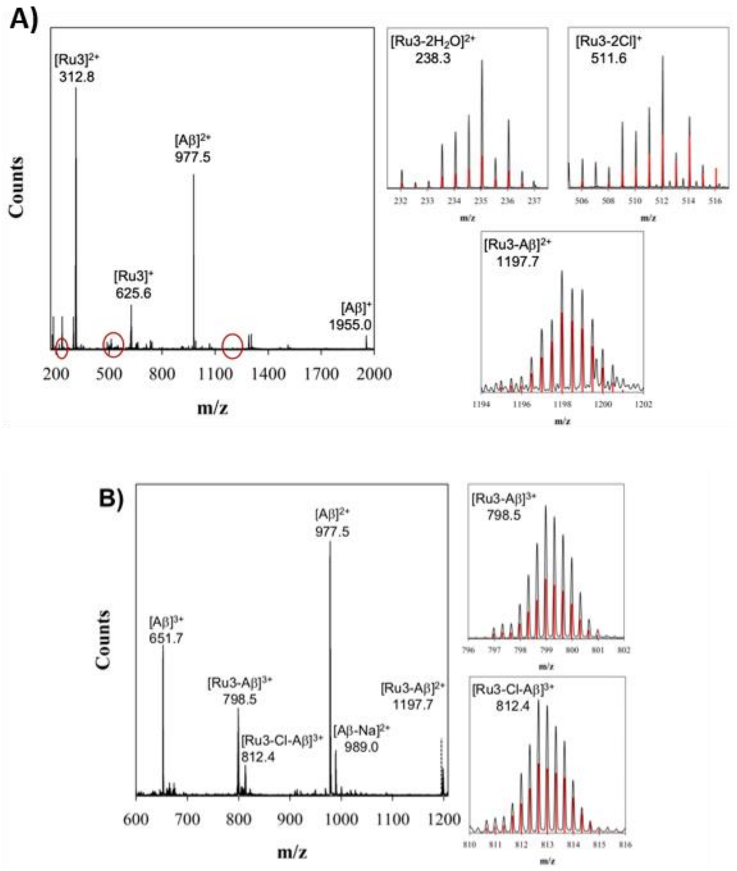

Figure S10. (A) ESI-MS of unactivated **Ru3** + $A\beta_{1-16}$ showing evidence of adduct formation. (B) ESI-MS of photoactivated **Ru3** + $A\beta_{1-16}$ also showing evidence of adduct formation. Zoomed regions exhibit the isotopic pattern of the adducts detected, and in red the theoretical isotopic pattern for the corresponding adduct. Samples were prepared in NH_4CO_3 buffer (20 mM, pH 9.0) and data was collected after 25 min. of activation.

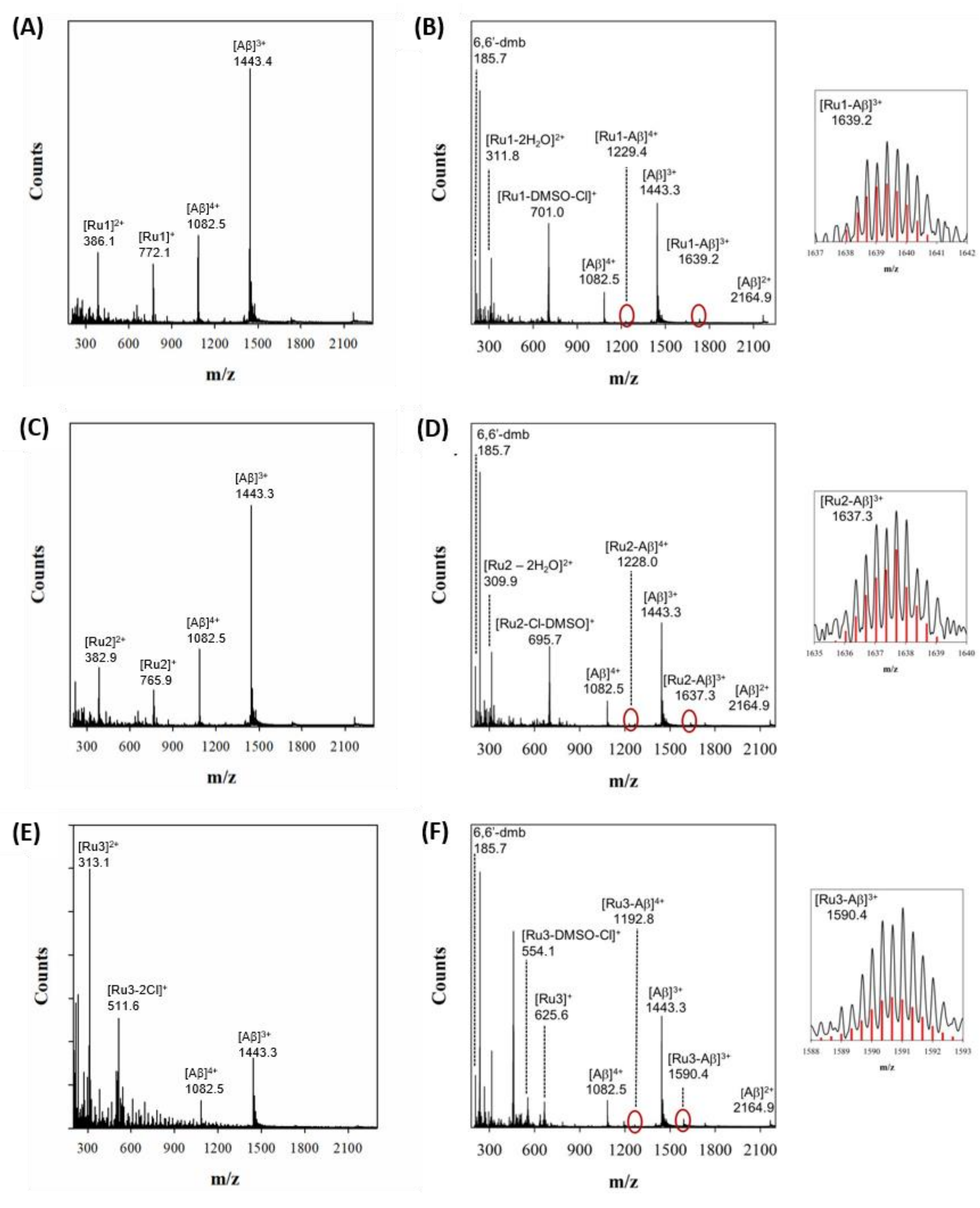

Figure S11. ESI-MS of **Ru1** (A), **Ru2** (C), and **Ru3** (E) and $A\beta_{1-40}$ in the absence of photoactivation. ESI-MS of **Ru1** (B), **Ru2** (D), and **Ru3** (F) and $A\beta_{1-40}$ after photoactivation indicating the adduct formation in the zoomed in regions. In red the theoretical isotopic pattern. Samples were prepared in NH_4CO_3 buffer (20 mM, pH 9.0) and data was collected after the respective times for activation.

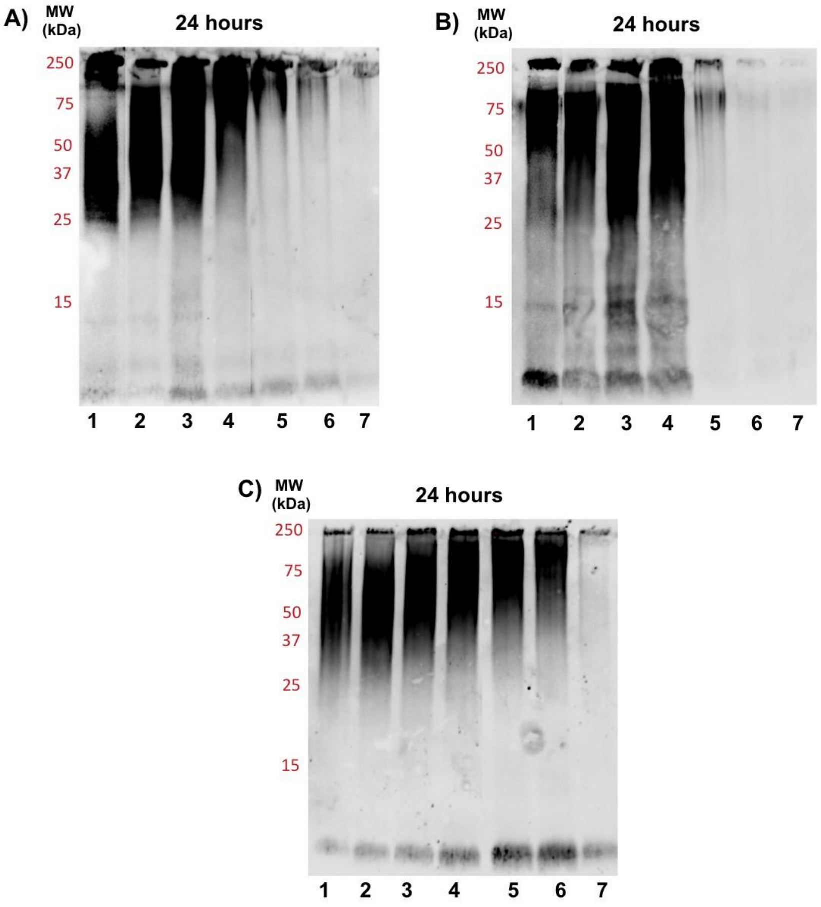

Figure S12. Gel Electrophoresis/Western blot of 25 μM Aβ_{1-42} and different concentrations of **Ru1** (A), **Ru2** (B), and **Ru3** (C) in PBS buffer (0.01 M, pH 7.4) at 24 h incubation with agitation at 37 °C, using anti-Aβ antibody 6E10. Lane 1: Aβ_{1-42}; lane 2: Aβ_{1-42} + 0.10 eq. Ru complex; lane 3 Aβ_{1-42} + 0.25 eq. Ru complex; lane 4: Aβ_{1-42} + 0.50 eq. Ru complex; lane 5: Aβ_{1-42} + 1.0 eq. Ru complex; lane 6: Aβ_{1-42} + 1.5 eq. Ru complex; lane 7: Aβ_{1-42} + 2.0 eq. Ru complex.

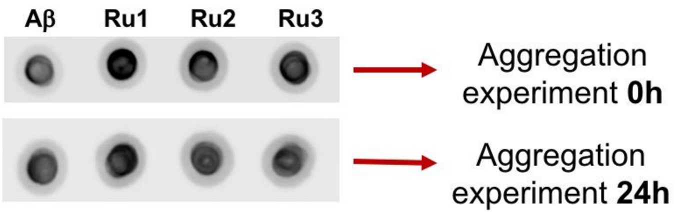

Figure S13. Dot blot of 25 μM Aβ_{1-42} alone, and 25 μM Aβ_{1-42} in the presence of 1.0 eq. of photoactivated **Ru1-3** at 0 h and 24 h of incubation showing that even after interaction with Ru complexes, the peptide is recognized by the 6E10 antibody.

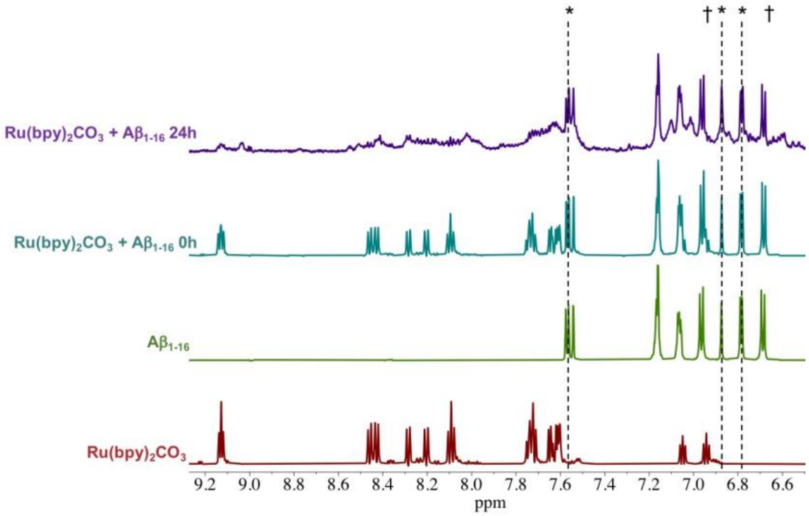

Figure S14. ^{1}H NMR spectra of Aβ_{1-16} (200 μM) in the presence of 1.0 eq. $Ru(bpy)_2CO_3$ showing no changes of peptide residues after 24 h of incubation. Samples were prepared in PBS buffer (0.01 M, pH 7.4) at 37 °C. * His^6, His^{13} and His^{14}. † Tyr^{10}. Broadening of signals is likely due to precipitation and/or multiple Ru species at the 24 h timepoint.

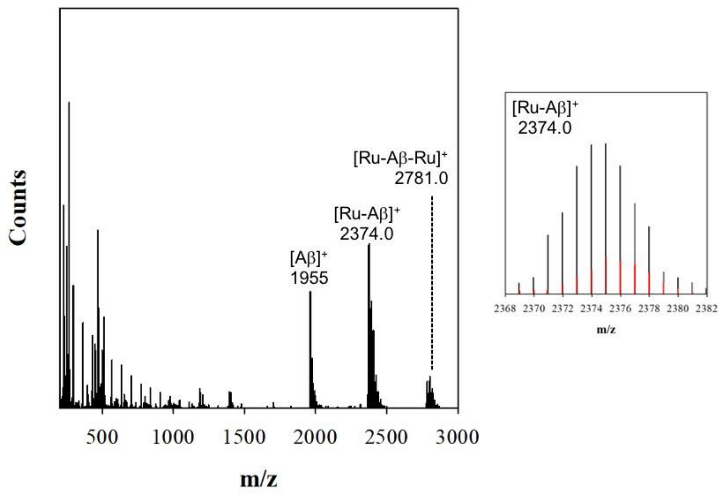

Figure S15. ESI-MS of $Ru(bpy)_2CO_3$ and $A\beta_{1-16}$ indicating adduct formation in the zoomed in region. The theoretical isotopic pattern is shown in red. Samples were prepared in NH_4CO_3 buffer (20 mM, pH 9.0) and data was collected after 25 min. activation time.

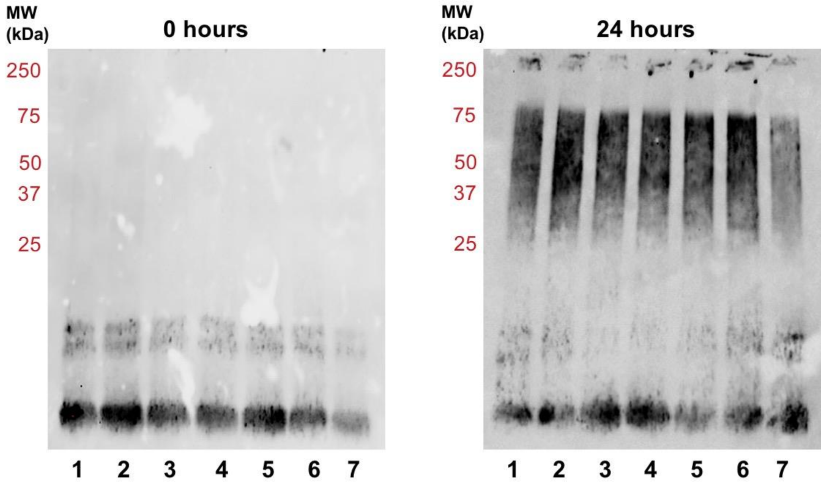

Figure S16. The Gel Electrophoresis/Western blot of 25 μM $A\beta_{1-42}$ and different concentrations of $Ru(bpy)_2CO_3$ in PBS buffer (0.01 M, pH 7.4) at 0 h and 24 h incubation with agitation at 37 °C, using anti-Aβ antibody 6E10. Lane 1: $A\beta_{1-42}$; lane 2: $A\beta_{1-42}$ + 0.10 eq. Ru complex; lane 3 $A\beta_{1-42}$+ 0.25 eq. Ru complex; lane 4: $A\beta_{1-42}$+ 0.50 eq. Ru complex; lane 5: $A\beta_{1-42}$+ 1.0 eq. Ru complex; lane 6: $A\beta_{1-42}$+ 1.5 eq. Ru complex; lane 7: $A\beta_{1-42}$+ 2.0 eq. Ru complex.

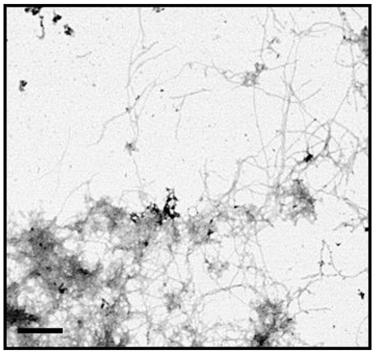

Figure S17. Increased magnification TEM image of amorphous aggregates and developing fibrils after incubation of $A\beta_{1-42}$ alone for 24 h (scale bar = 100 nm). See Figure S19 for further development into mature fibrils at 96 h.

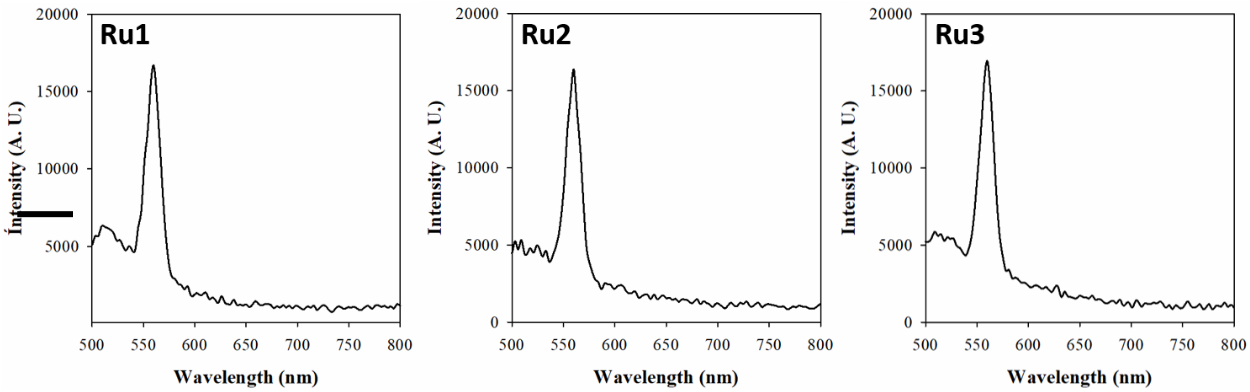

Figure S18. Emission Spectra of 10μM **Ru1-3** in pH 7.4 PBS buffer (0.01M). λ_{ex} = 470 nm, Water emission line at 558 nm.

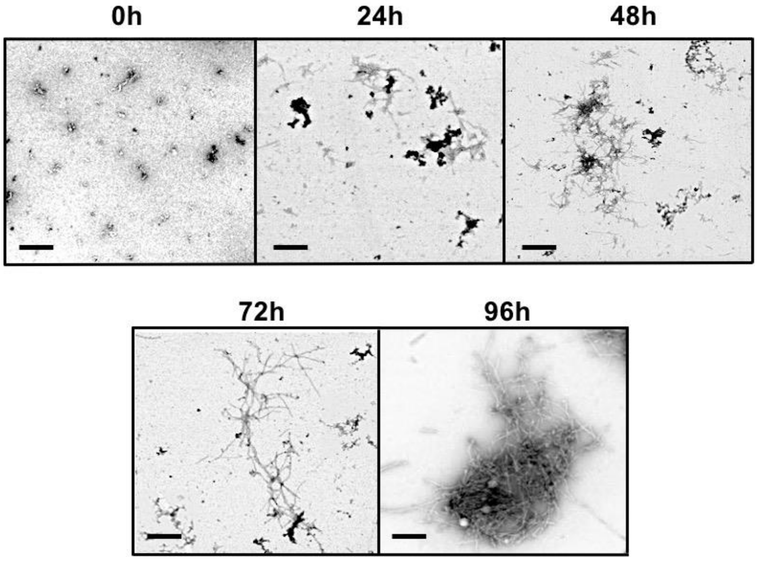

Figure S19. TEM images of the change in morphology of Aβ_{1-42} over time leading to the formation of mature fibrils after 96 h of incubation (scale bar = 200nm).

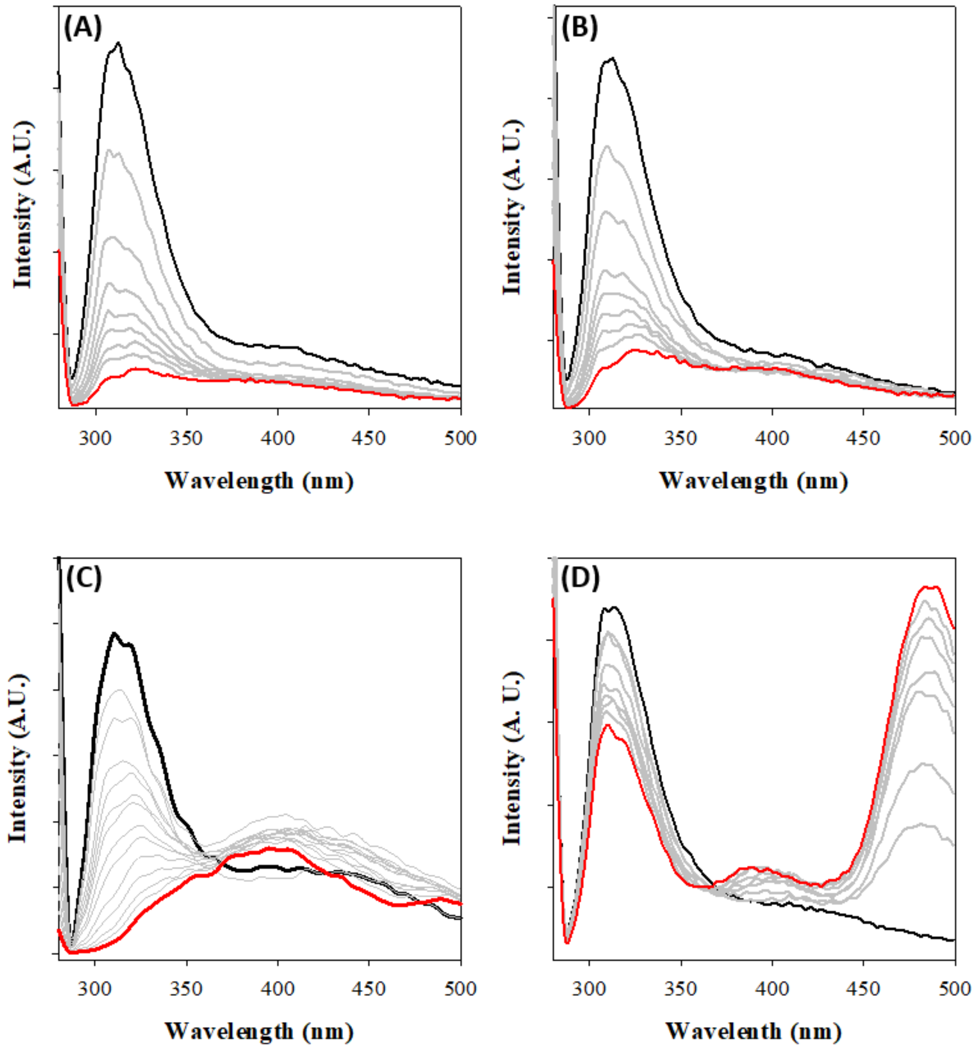

Figure S20. Aβ_{1-42} fibril tyrosine fluorescence changes upon addition of **Ru1-3** and ThT. (A) **Ru1** 0 (black) to 2 (red) eq. (B) **Ru2** 0 (black) to 2 (red) eq. (C) **Ru3** 0 (black) to 8 (red) eq. (D) ThT 0 (black) to 2 (red) eq. Conditions: Addition of **Ru1-3** and ThT to Aβ_{1-42} fibrils 10 μM in PBS (0.01 M, pH 7.4). Fluorescence intensity was measured immediately ($\lambda_{ex}/\lambda_{em}$ = 275/310 nm).

Table S1. Relative docking score of **Ru1-3** at 2MXU (A-G) and 5OQV (H-Q) binding sites (kcal/mol).

			2MXU		
**Site**	**Ru1**	**Ru2**	**Ru3**	**δ(Ru3 - Ru1)**	**δ(Ru3 - Ru2)**
**A**	-12.8	-13.1	-5.19	7.65	7.96
**B**	-5.80	-5.77	-3.52	2.28	2.25
**C**	-10.0	-10.9	-4.88	5.13	6.01
**D**	-14.4	-15.0	-14.3	0.16	0.75
**E**	-15.1	-15.1	-14.9	0.23	0.24
**F**	-16.6	-16.6	-18.3	-1.65	-1.67
**G**	-15.5	-16.7	-8.3	7.23	8.44
			**5OQV**		
**H**	-14.6	-14.9	-13.3	1.37	1.64
**I**	-9.08	-9.84	-14.9	-5.87	-5.11
**J**	-9.80	-11.2	-3.63	6.17	7.58
**K**	-21.1	-21.4	-18.0	3.15	3.47
**L**	-15.7	-16.0	-14.5	1.27	1.55
**M**	-11.5	-11.9	-9.25	2.22	2.65
**N**	-11.3	-11.4	-4.60	6.75	6.83
**O**	-10.3	-10.5	-12.3	-2.01	-1.81
**P**	-15.6	-15.5	-12.2	3.35	3.29
**Q**	-18.6	-18.5	-18.4	0.26	0.15

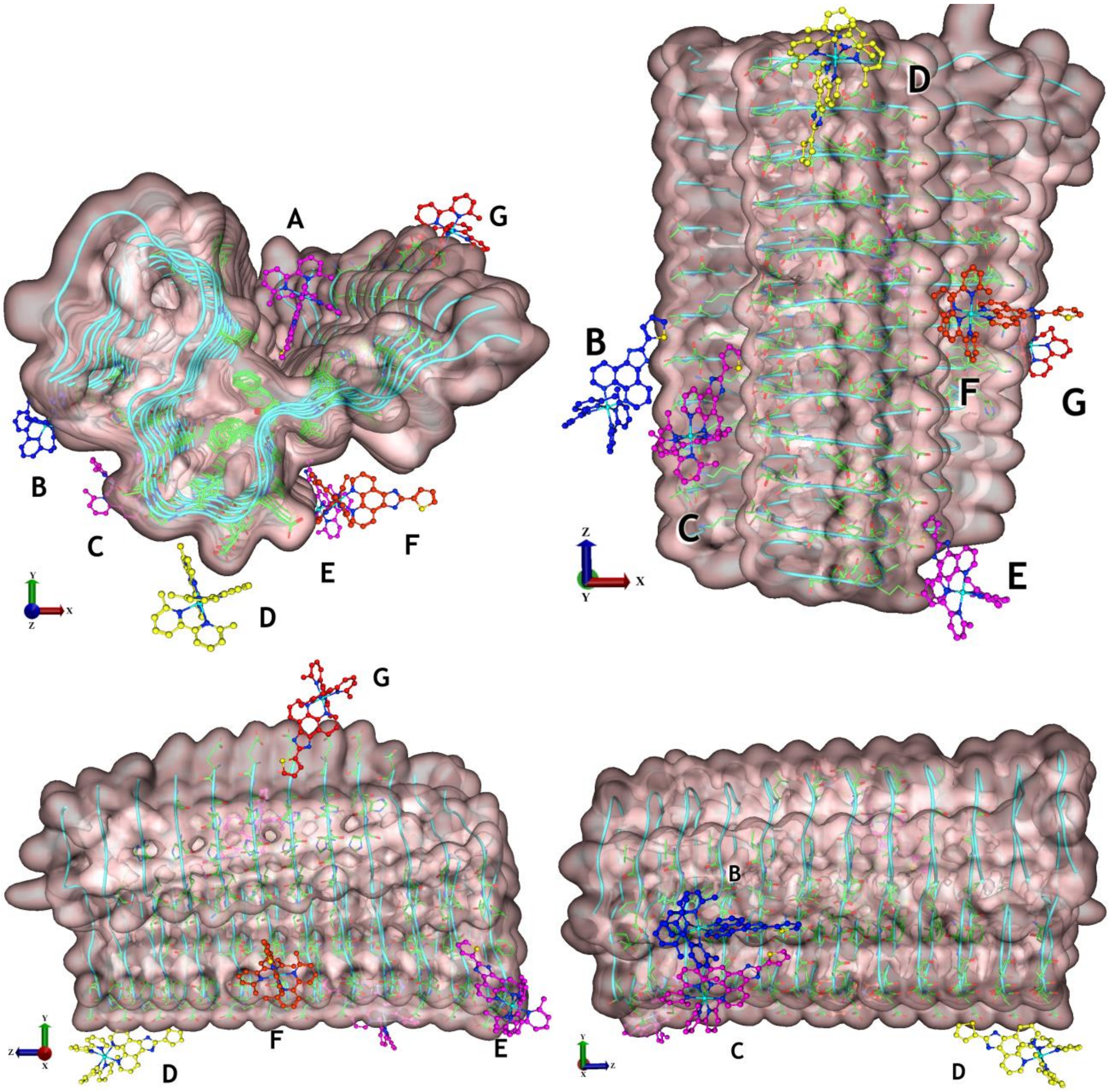

Figure S21: Binding sites found for **Ru1-3** on 2MXU. Images are rotated in 3 dimensions to present the whole surface. For clarity, only **Ru1** results are shown, as **Ru2** is essentialy identical. Zoomed views of each site are provided below (Figure S23)**.** We excluded sites with a ligand interaction (docking score) below 5 kcal/mol (See Table S1 for values).

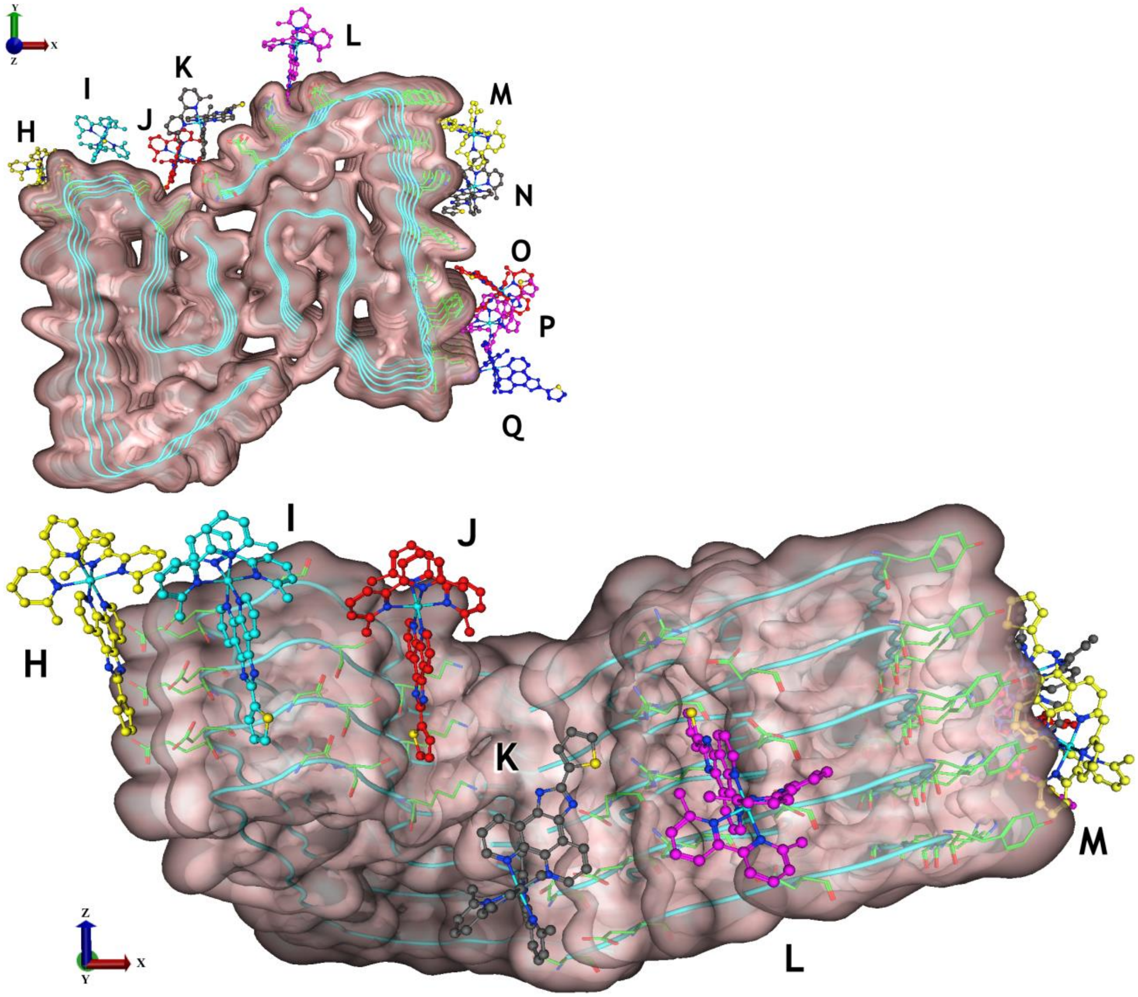
L
K
I
J
H
M
N
O
P
Q
I
J
H
K
M
L

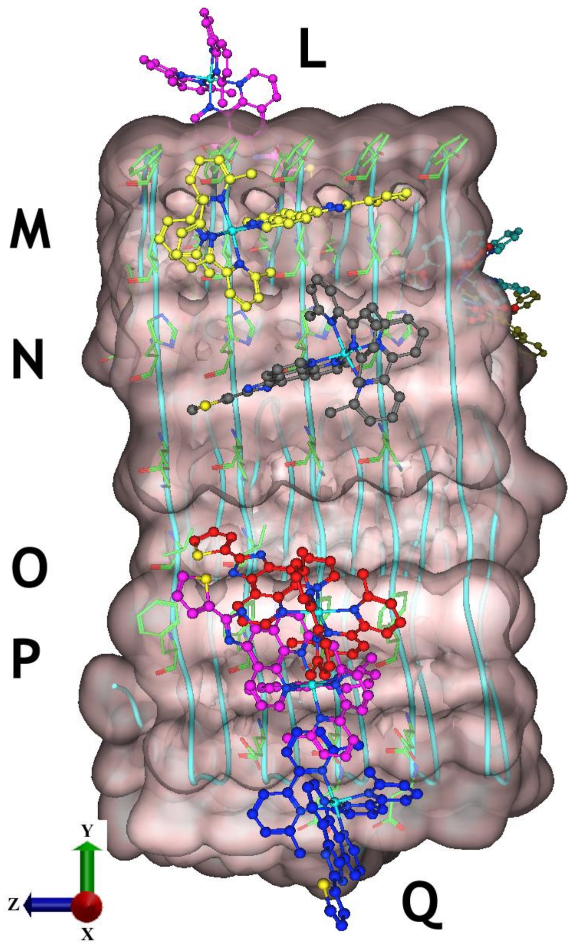

Figure S22: Binding sites found for **Ru1-3** on 5OQV. Images are rotated in 3 dimensions to present the whole surface. For clarity, only **Ru1** results are shown, as **Ru2** is essentialy identical. Zoomed views of each site are provided below (Figure S23**)**. As a dimer, binding sites on the other monomer were essentially identical and omitted for clarity.

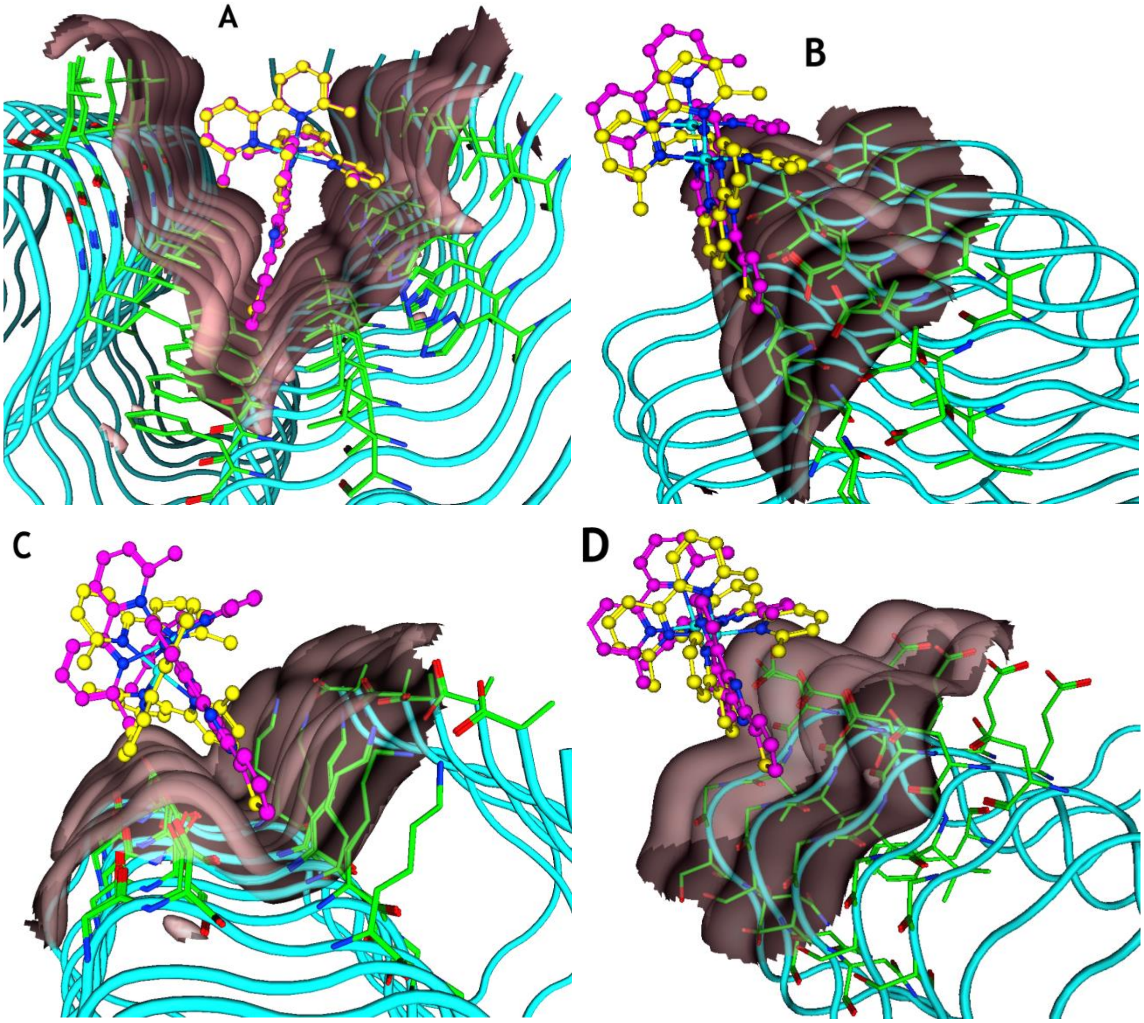
A
B
C
D

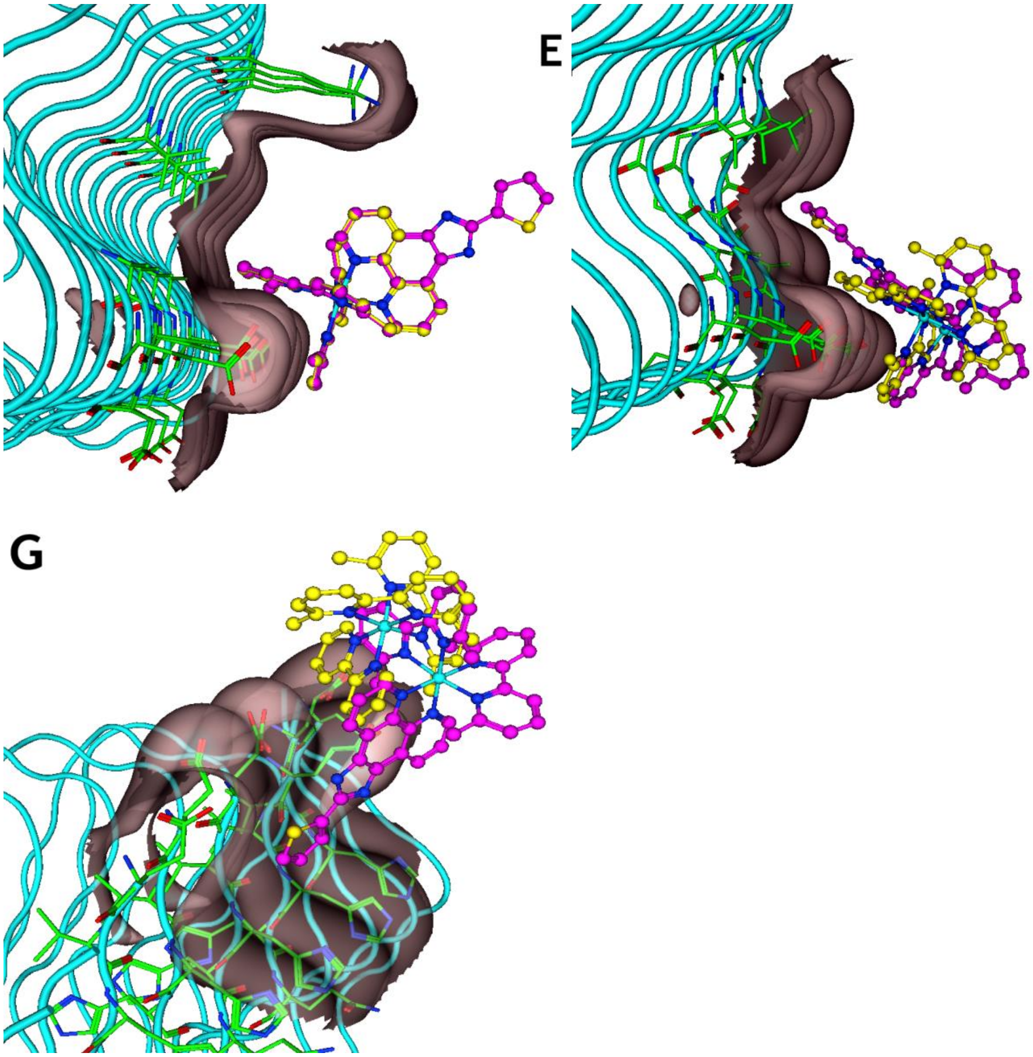
E
F
G

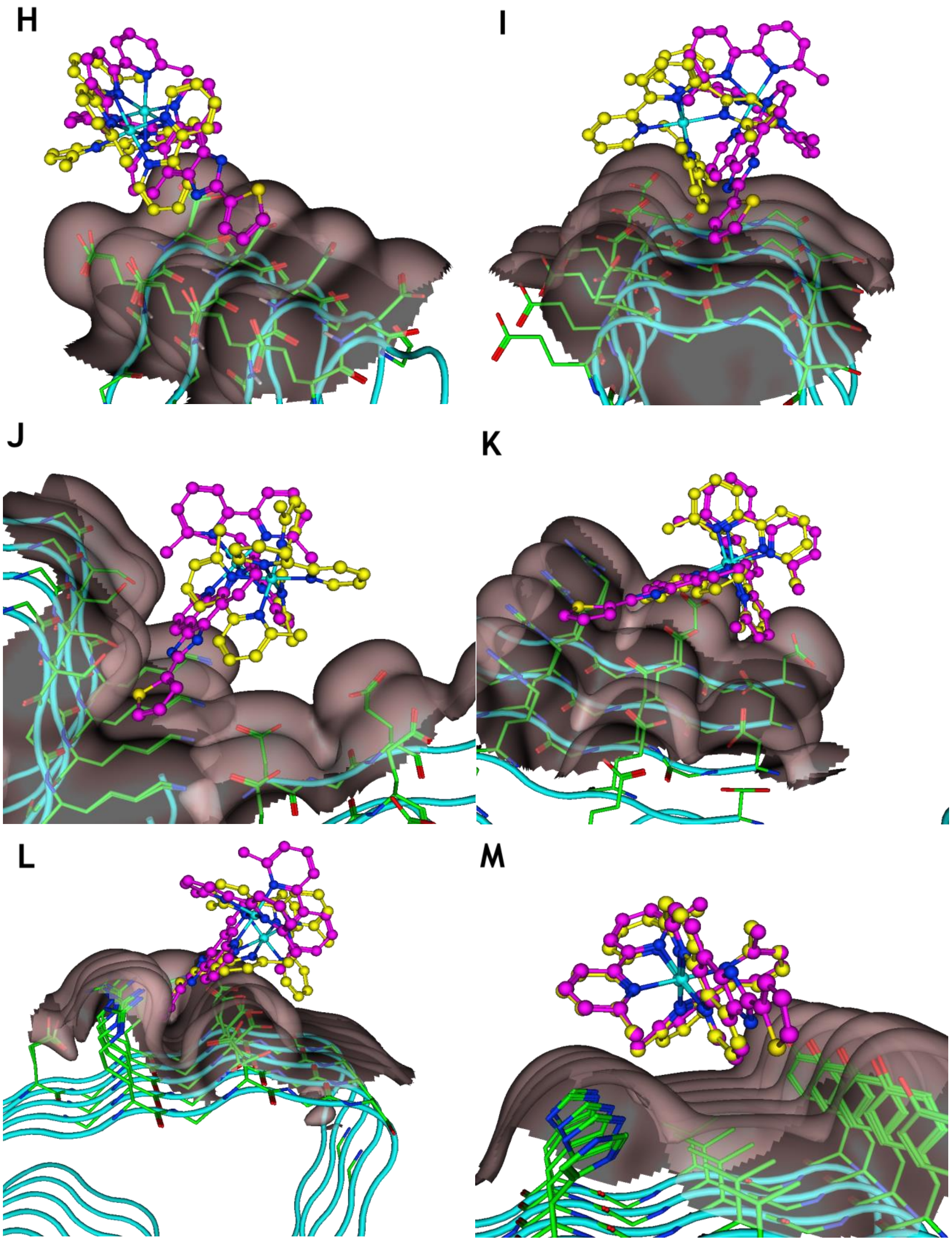
H
I
J
K
L
M

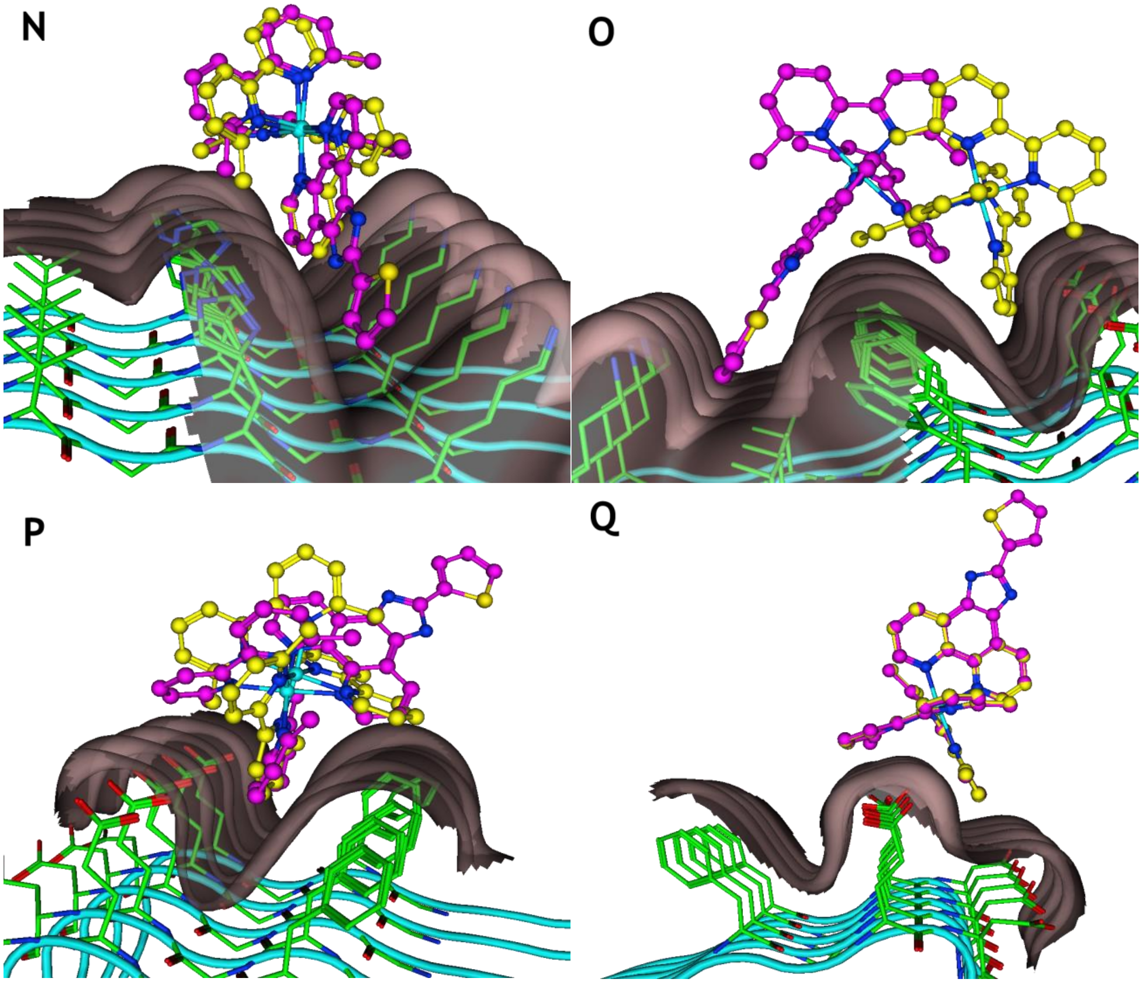

Figure S23: Comparison of **Ru1** (purple carbons) and **Ru3** (yellow carbons) in each identified binding site in PDB structures 2MXU (sites A – G) and 5OQV (sites H-Q). The docking describes a shallow potential energy surface, with multiple bindings sites effectively contributing. Particular attention is drawn to sites F, I, and O as clearly demonstrating cases where **Ru3** appears to bind more strongly due to a more accessible metal center available to carboxylate interactions. Other sites (e.g. B – D, H, J, L, P) show a shift in **Ru3** position, but are equal or lower in score.

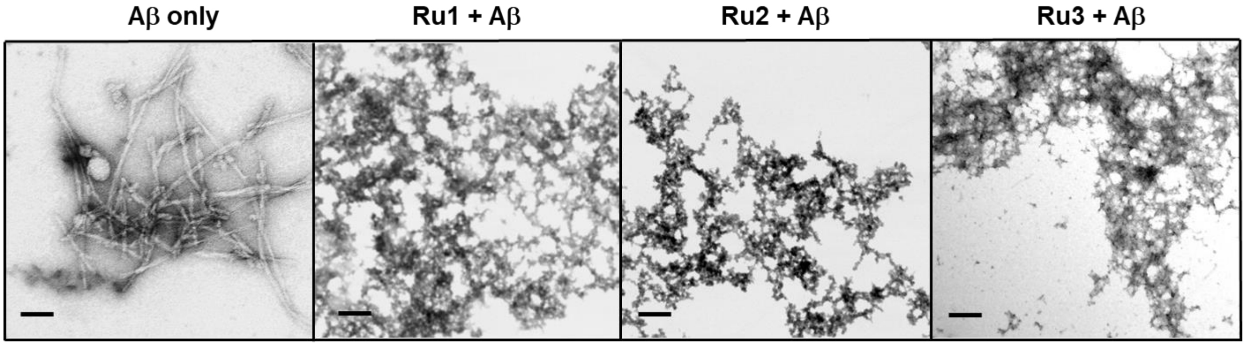

Figure S24. Increased magnification TEM images investigating the influence of 1.0 eq. of Ru1-3 on the morphology of fibrillar $A\beta_{1-42}$ (25 μM) at 24 h for photoactivated samples. (scale bar = 100 nm).

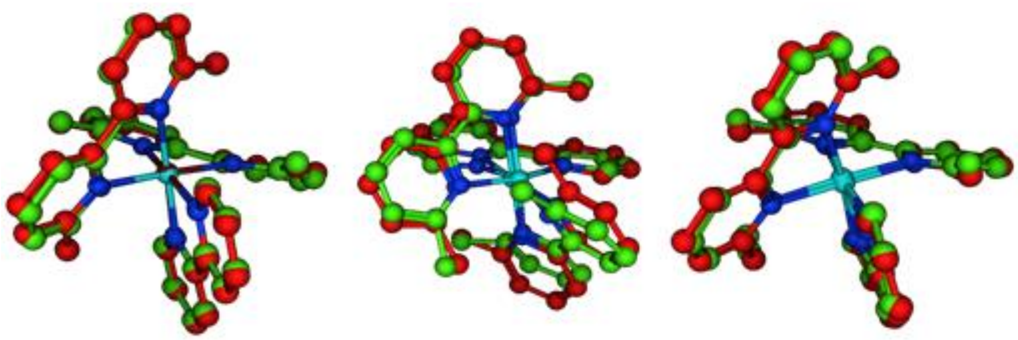

Figure S25. **Left**: Ru(II)-3 (red carbons B3LYP/LANL2DZ) and Fe(II)-3 (green carbons B3LYP/LANL2DZ) aligned. **Middle**: Ru(II)-3 (B3LYP/LANL2DZ, red carbons) and Ru(II)-3 (AMBER, Green carbons) aligned. **Right**: Ru(II)-3 (B3LYP/LANL2DZ, red carbons) and Ru(II)-3 (MMFF94x, Green carbons) aligned. Whilst the left and right images show minimal differences in overall geometry, stark changes are observed in the middle image, indicating that MMFF94x is better at reproducing the geometries of the Ru(II) complexes from DFT.

<u>References:</u>

1. Sainuddin, T.; Pinto, M.; Yin, H.; Hetu, M.; Colpitts, J.; McFarland, S. A., *J. Inorg. Biochem.* **2016,** *158,* 45-54.
2. Roque III, J.; Havrylyuk, D.; Barrett, P. C.; Sainuddin, T.; McCain, J.; Colón, K.; Sparks, W. T.; Bradner, E.; Monro, S.; Heidary, D.; Cameron, C. G.; Glazer, E. C.; McFarland, S. A., *Photochem. Photobiol.* **2020,** *96* (2), 327-339.
3. Cuello-Garibo, J.-A.; Pérez-Gallent, E.; van der Boon, L.; Siegler, M. A.; Bonnet, S., *Inorg. Chem.* **2017,** *56* (9), 4818-4828.
4. Sabaté, R.; Gallardo, M.; Estelrich, J., *Biopolymers* **2003,** *71* (2), 190-5.
5. Pachahara, S. K.; Chaudhary, N.; Subbalakshmi, C.; Nagaraj, R., *J. Pept. Sci.* **2012,** *18* (4), 233-241.
6. Guilloreau, L.; Combalbert, S.; Sournia-Saquet, A.; Mazarguil, H.; Faller, P., *Chembiochem* **2007,** *8* (11), 1317-25.
7. Howerton, B. S.; Heidary, D. K.; Glazer, E. C., *J. Am. Chem. Soc.* **2012,** *134* (20), 8324-8327.
8. Wachter, E.; Heidary, D. K.; Howerton, B. S.; Parkin, S.; Glazer, E. C., *Chem. Commun.* **2012,** *48* (77), 9649-9651.
9. Wachter, E.; Howerton, B. S.; Hall, E. C.; Parkin, S.; Glazer, E. C., *Chem. Commun.* **2014,** *50* (3), 311-3.
10. Hidayatullah, A. N.; Wachter, E.; Heidary, D. K.; Parkin, S.; Glazer, E. C., *Inorg. Chem.* **2014,** *53* (19), 10030-2.
11. Jones, M. R.; Service, E. L.; Thompson, J. R.; Wang, M. C. P.; Kimsey, I. J.; DeToma, A. S.; Ramamoorthy, A.; Lim, M. H.; Storr, T., *Metallomics* **2012,** *4* (9), 910-920.
12. Mancino, A. M.; Hindo, S. S.; Kochi, A.; Lim, M. H., *Inorg. Chem.* **2009,** *48* (20), 9596-9598.
13. Wang, X.; Wang, X.; Zhang, C.; Jiao, Y.; Guo, Z., *Chem. Sci.* **2012,** *3* (4), 1304-1312.
14. Frisch, M. J., et al., *Gaussian 16, Revision C.01*. Gaussian, Inc.: Wallingford, CT, 2016.
15. Cornell, W. D.; Cieplak, P.; Bayly, C. I.; Gould, I. R.; Merz, K. M.; Ferguson, D. M.; Spellmeyer, D. C.; Fox, T.; Caldwell, J. W.; Kollman, P. A., *J. Am. Chem. Soc.* **1995,** *117* (19), 5179-5197.
16. Jakalian, A.; Jack, D. B.; Bayly, C. I., *J. Comput. Chem.* **2002,** *23* (16), 1623-1641.
17. Halgren, T. A., *J. Comput. Chem.* **1996,** *17* (5-6), 490-519.
18. Corbeil, C. R.; Williams, C. I.; Labute, P., *J. Comput. Aided Mol. Des.* **2012,** *26* (6), 775-786.
19. Li, Q.; Folly da Silva Constantino, L.; Spies, M. A., Chapter Eleven - Integrating Experimental and In Silico HTS in the Discovery of Inhibitors of Protein–Nucleic Acid Interactions. In *Methods Enzymol.*, Spies, M.; Malkova, A., Eds. Academic Press: 2018; Vol. 601, pp 243-273.
20. Wadood, A.; Riaz, M.; Jamal, S. B.; Shah, M.; Lodhi, M. A., *Bioinformation* **2013,** *9* (6), 309-314.
